# The contribution from psychological, social, and organizational work factors to risk of disability retirement: a systematic review with meta-analyses

**DOI:** 10.1186/s12889-017-4059-4

**Published:** 2017-02-08

**Authors:** Stein Knardahl, Håkon A. Johannessen, Tom Sterud, Mikko Härmä, Reiner Rugulies, Jorma Seitsamo, Vilhelm Borg

**Affiliations:** 1 Department of work psychology and physiology, National Institute of Occupational Health, Oslo, Norway; 2 Department of Occupational Health Surveillance, National Institute of Occupational Health, Oslo, Norway; 3 Finnish Institute of Occupational Health, Helsinki, Finland; 4 National Research Centre for the Working Environment, Copenhagen, Denmark; 5 Department of Public Health, University of Copenhagen, Copenhagen, Denmark; 6 Department of Psychology, University of Copenhagen, Copenhagen, Denmark

**Keywords:** Disability retirement, Disability pension, Work, Psychological, Social, Psychosocial, Organizational, Shift, Systematic review

## Abstract

**Background:**

Previous studies indicate that psychological, social, and organizational factors at work contribute to health, motivation, absence from work, and functional ability.

The objective of the study was to assess the current state of knowledge of the contribution of psychological, social, and organizational factors to disability retirement by a systematic review and meta-analyses.

**Methods:**

*Data sources*: A systematic literature search for studies of retirement due to disability in Medline, Embase, and PsychINFO was performed. Reference lists of relevant articles were hand-searched for additional studies. *Data extraction*: Internal validity was assessed independently by two referees with a detailed checklist for sources of bias. Conclusions were drawn based on studies with acceptable quality. *Data synthesis*: We calculated combined effect estimates by means of averaged associations (Risk ratios) across samples, weighting observed associations by the study’s sample size. Thirty-nine studies of accepted quality were found, 37 of which from the Nordic countries.

**Results:**

There was moderate evidence for the role of low control (supported by weighted average RR = 1.40; 95% CI = 1.21-1.61) and moderate evidence for the combination of high demands and low control (although weighted average was RR = 1.45; 95% CI = 0.96-2.19) as predictors of disability retirement. There were no major systematic differences in findings between the highest rated and the lowest rated studies that passed the criterion for adequate quality. There was limited evidence for downsizing, organizational change, lack of employee development and supplementary training, repetitive work tasks, effort-reward imbalance to increase risk of disability pension. Very limited evidence was found for job demands, evening or night work, and low social support from ones superior.

**Conclusions:**

Psychological and organizational factors at work contribute to disability retirement with the most robust evidence for the role of work control. We recommend the measurement of specific exposure factors in future studies.

**Electronic supplementary material:**

The online version of this article (doi:10.1186/s12889-017-4059-4) contains supplementary material, which is available to authorized users.

## Background

With the unprecedented global population ageing, extending the working life is becoming increasingly important for sustaining welfare for all citizens [1]. Early exit from working life due to disability incurs large production losses and compensations costs for societies as well as challenges to the quality of life to the individuals. The objective of the present systematic review was to assess the state of knowledge of the contribution of psychological, social, and organizational factors at work to retirement due to disability.

Disability is defined as the general inability to perform ones job, either due to (i) a problem in body function or structure (impairment), (ii) difficulties in executing tasks (activity limitation), or (iii) problems experienced in participating in tasks or social relations at the workplace (participation restriction), cfr The International Classification of Functioning, Disability, and Health (ICF; http://www.who.int/classifications/icf/en/) developed by the WHO. While job disability is a condition or state at which one decides that a job cannot be performed, the level of ‘work ability’ may vary over time. Work ability is a central concept in social security and rehabilitation medicine referring to abilities necessary to perform and hold a job. Competence is a central concept in management and employment referring to knowledge and skills and relevant abilities. Hence, these concepts overlap (Tengland [2]) and both are either defined relative to demands posed by one specific job (e.g., the job held by an individual) or relative to demands of holding a job in general [2].

A large body of studies have shown that psychological and social factors at work may contribute to health and disease. Most of these studies have tested predictions of the demand-control model (Karasek [3]). Originally this “model predicts that mental strain results from the interaction of job demands and decision latitude” [3]. This model has been paramount in advancing research from the ambiguous and circular concept “work stress” to elucidation of the exposure dimensions demands and control that relate to basic research on responding to challenge (e.g., Weiss [4]). An instrument developed to measure these factors, the Job Content Questionnaire (JCQ) has been widely used in research of work and health [5]. The combination of high level of demand and low levels of control seems to contribute to cardiovascular disease (e.g., Kivimäki et al [6]) and several other health problems (e.g., Kraatz et al [7]).

The demand-control model addresses broad dimensions, but still only represents few aspects of exposures at work. Recent research of work and health has provided knowledge of other factors; for example effort-reward imbalance [5], organizational justice (e.g., Kivimäki et al [8]) and Ylipaavalniemi et al [9]), team climate (e.g., Ylipaavalniemi et al. [9]) [10], interpersonal conflict [11]”[12], and role conflict (Christensen & Knardahl [13, 14]).

Of many organizational factors, downsizing, organization of work schedules (shift work, and long working hours) have been reported to contribute to health and disease (e.g., heart disease [15–17] and musculoskeletal pain disorders [18]. Rapid rates of upsizing may also contribute to health problems [19].

The combination of illness perceptions, illness beliefs, and the appraisal of demands posed by the work tasks influence an individual’s appraisal of work ability. Subjective appraisal of work ability may influence attitudes to work. The workplace is an arena where individuals face challenges from work tasks and social interactions. Work also provides opportunities for positive achievement, fulfilment, and friendship. For many people the job is a major source of feedback on attitudes and behaviour. Studies of organizational psychology have revealed psychological factors of significance to work motivation (e.g., Hackman & Oldham [20]) and Adams [21] and global satisfaction with ones job (e.g., Spector [22]). Hypothetically, psychological, social, and organizational factors at work may contribute to early retirement with disability pension by influencing several of the processes leading from a state of good health and work ability to a state of reduced health and disability.

Ilmarinen, Tuomi, and Seitsamo [23] proposed that several dimensions of health resources, competence, values, and factors at work contributes to work ability and modelled this like a house of four floors (“the house model of work ability”). The theoretical basis of the present study was three assumptions. (I) retirement due to disability is a result of a series of processes, each with multifactorial causation. (II) Both biological/medical, psychological, and social factors contribute in these processes: clinical medical condition, physiological and cognitive function, competence, job demand characteristics, individual appraisal of work ability, physician’s assessment of work ability, job motivation, and attitudes to one’s job may contribute in the processes leading from high work ability, and adequate competence to disability resulting in exit from working life (e.g., de Wind et al. [24]; Volanen et al. [25]). (III) Psychological, social, and organizational factors at work influence several of these factors and processes and henceforth may contribute to retirement due to disability. Society-level factors influence exit from working life and retirement compensation, but are outside the scope of the present study.

The present systematic review aimed to answer the following research questions: Which psychological task-level work factors contribute to retirement due to disability? Which social interaction factors at work contribute to retirement due to disability? Which organizational work factors contribute to retirement due to disability? We did not limit the review to models, theories, or to specific factors and sought to grade the level of evidence for each factor studied.

## Methods

With the aim to examine whether psychological, social, or organizational factors at work contribute to retirement due to disability, we performed systematic literature searches plus an extensive evaluation of the methodological quality of the retrieved articles. Retirement was defined as permanently not performing paid work. In order to include any relevant work factor and allow variations in wording of factors (constructs), searches did not specify work factors. We also performed meta-analyses when applicable.

Methods of inclusion criteria, analyses, and eligibility were specified in an unpublished protocol (developed by the research group to ensure that all procedures were standardized and adhered to throughout the study). Minor modifications of protocols were performed during the study. All modifications were documented and all conclusions were based on the final version of the methods.

### Eligibility criteria and search strategy

Disability retirement was defined as permanently not performing paid work due to disability. *Psychological factors* were defined as variables pertaining to the contents of a job and work tasks. *Social factors* were defined as interactions with other people, either co-workers, superiors/leaders, or clients, customers, or patients. We defined *organizational factors* as ways work is organized, e.g., working hours and shift-work systems, downsizing, upsizing, reorganization e.g., merging of units.

For inclusion, studies had to meet all the following criteria:

1. *Outcome measures*: addressed registry-based disability pension awards or self-reported retirement from work due to ill health or disease;
2. *Types of exposures*: measured any organizational, psychological, and social exposure pertaining to work in subjects that were employed and working.;
3. *Types of studies*: designed as a prospective cohort study, case control study (longitudinal), or intervention study;
4. *Types of participants*: employees, reported analyses estimating effects of work factors.

The review was limited to publications written in English, German, Danish, Finnish, Norwegian, or Swedish.

#### Information sources

We searched systematically Medline, Embase and PsycInfo up to April 23^rd^, 2015 to identify primary studies that addressed the risk of retirement due to disability in relation to any organizational, psychological, and social exposure pertaining to work.

An additional table A (see Additional file 1, Table A: Search strategy) shows the search strategy that was developed and adapted for each database with a combination of free text terms and controlled, hierarchical vocabulary (e.g., Medical Subject Heading terms for Medline). No limits and a search strategy with a high sensitivity were selected. The search terms were constructed to identify articles that addressed the risk of disability pension awards or related outcomes pertaining to retirement, independent of work-related exposures. We tested the specificity and sensitivity of each eligible search term before inclusion in the search string. Pilot searches showed that search profiles with exposures terms (psychosocial, demands, control, etc) did not result in more relevant sets of studies and often excluded relevant studies.

#### Screening

Two reviewers retrieved and screened the 19545 abstracts produced by the searches. When in doubt, the study was read in full text. The full-text versions of all potentially relevant articles were independently reviewed for inclusion by two of the authors. If disagreement or doubt, the article was subjected to formal assessment of methodological quality.

In addition to database searches, the reference lists of all articles of acceptable quality were inspected (“hand-searched”). We found one additional article on factors determining remaining at work [26], but decided that the definition of “remaining at work” did not meet our inclusion criteria of retirement due to disability.

The current review defined psychological, social, and organizational factors at work as *exposures* that individuals are subjected to during work. Studies of personality traits were not relevant for the present review. It may be argued that job dissatisfaction, low commitment, low job involvement may be proxies of poor work environments. However, these factors are mediators between exposures and outcomes, not exposures. Therefore, a study which investigated effects of job satisfaction and job enjoyment [27] and a study of organizational commitment and meaning of work [28] were excluded from the systematic review.

Data on health status at baseline was scored as a potential confounder in the quality check list (positive if measured and adjusted or stratified in analyses). Some studies of prognostic factors in specific diseases like insomnia, obesity, rheumatoid arthritis or coronary heart disease have measured work factors as predictors of disability retirement [29–33]. However, many of these studies have treated work factors as covariates only [29–31].

### Data extraction

We extracted data from each included study using the following variables: study characteristics (Authors’ of the study, date of the study, and study location), exposures investigated (instruments used to measure factors at work), employee groups/types of work and number of subjects studied, outcomes/definition of disability pension and number of cases, effect estimates (the most completely adjusted estimates reported), and confounders controlled for.

### Risk of bias: The assessment of validity of findings (study quality)

The present systematic critical review defined the quality of primary studies as *internal validity*, the extent to which the effects reported in a study are truly caused by the treatment or exposure in the study sample (rather than being due to other biasing effects of extraneous variables). *External validity* (generalizability) determines which (specific) populations the conclusions apply to.

Systematic reviews have assessed methodological quality of primary studies by several systems. The Grading of Recommendations Assessment, Development and Evaluation Working Group (GRADE) system for the evaluation of treatment trials grade evidence as high (GRADE 4), moderate (3), limited (2), and very limited (1) [34]. In studies of treatments the serious threats to internal validity of conclusions are selection bias and information bias due to inadequate blinding. There is no consensus or gold standard for assessing the quality of observational epidemiological studies (e.g., Sanderson et al. [35]; Shamliyan et al. [36]). Recommendations for reporting or evaluating observational studies (e.g., the STROBE statement) [37] address variables in general terms: “give sources of data and details of methods of assessment (measurement)”. The GRADE system categorizes observational studies as limited evidence (GRADE 2) even if conducted with prospective design and no known selection bias because of high risk of bias [36, 38]. However, the evidence may be upgraded to moderate (GRADE 3) if several studies show the same result or if a limited number of studies are unequivocal.

Most studies of psychological and social work exposures are based on self-reported data which present challenges to validity of measurement methods. The individual’s reporting is influenced by psychological mechanisms like perception, cognitive appraisal, expectancies, attitudes, etc, which in turn are influenced by personality traits and culture (e.g., Watson et al. [39]; Chen & Spector. [40]; Oliver et al. [41]). Recommendations for reporting or evaluating observational studies (Sanderson et al, 2007; the STROBE statement) [35, 37] do not address evaluating psychometrics of variables [42].

The present systematic review evaluated primary articles with a detailed check list (see Additional file 1, Table B: Quality assessment check list) which included items for grading quality of subjective-report methods. There were separate check lists for each study design type since some items are design specific (e.g., blinding and randomization in experimental designs). Since work exposure variables may vary considerably over time, single-point measurements may be unreliable estimates of exposure. Hence, an item addressing repeated measurements of exposures was included.

The main arguments for applying a detailed check list for observational studies were to ensure that (i) reviewers actively search out all information relevant to internal validity in each article, (ii) the two reviewers put equal weights on sources of bias, (iii) to provide a standard for grading methods based on different self-report instruments, observations, or registries, and to (iv) to provide full transparency of assessments. In addition to assessing internal validity (recruitment of study population/subjects; methods for exposure measurements; methods for outcome measurements; analysis and data presentation; and inclusion of confounders), we scored external validity (generalizability, the representativeness of the study population), and moderators (other types of exposures at work (e.g., physical, chemical) and leisure-time exposures). The two latter aspects are not taken into account in the present review. Before the scoring of articles took place, a pilot test of the check list was conducted by all reviewers to test the system.

Each study was first assessed independently by two reviewers. After assessing quality, the two referees compared and scored the study. If there was disagreement on checklist item scores, the referees discussed the reason for disagreement and agreed upon the score of the item. All authors participated in the assessment of quality.

The 27 different items of the checklist for internal validity of prospective studies were weighted for their potential significance for methodological quality (0-3 points). Factors of potential serious bias were assessed by more than one check-list item and higher obtainable scores. The grading of subjective-report methods for measuring exposures contained items pertaining to psychometric quality of instruments (explicit documentation of validity and reliability, repeated measurements) and reporting behavior (analysis of data at organizational unit-level), and reporting historical exposures. These are methodological measures that improve quality, but have not been considered necessary for accepting studies in epidemiology journals.

The scores were summed and a total score for *internal validity* was the basis for the conclusion of quality. To be given a maximum score (100%) a study must exhibit no discernible selection bias, attrition to follow-up lower than 15%, all measurements performed with objective (neutral) methods using interval or ratio scales, include three or more measurements of exposure factors (high reliability), include analyses which control confounders age, gender, education, socioeconomic gradients, and perform comprehensive statistical analyses. After having scored all articles published until 2012 the research group concluded that studies meeting customary criteria for acceptable methods exhibited scores exceeding 50% of maximum. The criterion for accepting methodological quality of a study was set to *internal validity* score of 50% or more. This level eliminated studies with “(1) failure to develop and apply appropriate eligibility criteria (inclusion of control population), (2) flawed measurement of both exposure and outcome (3) failure to adequately control confounding, and (4) incomplete follow-up” (GRADE guidelines) [38]. The highest score was 81% [43]. The study group agreed that studies that scored 66% or higher could be defined as high-quality studies. We have not found previous studies differentiating between acceptable and high quality base on detailed check list of all factors listed above.

*Conclusions* were based on studies with acceptable quality only. The conclusion “*high evidence* for an effect” required that randomized control studies of interventions targeted at a specific exposure factor (a change of exposure) showed that this exposure was significant. The conclusion “*moderate evidence*” required that there was sufficient reason to upgrade evidence from observational studies from the normal level of limited evidence: either (i) two or more observational studies of acceptable quality showed the same effect with no studies showing nonsignificant or opposite effects, or (ii) many observational studies of accepted quality showed an effect and in addition, a significant combined effect estimate in meta-analyses. The conclusion “*limited evidence*” was made if (i) there was only one study of acceptable quality of the factor in question (no replication) and this study showed a significant effect, or (ii) there were studies showing significant and a small number showing nonsignificant effects, but none showing significant opposite effects. The conclusion “*very limited evidence*” was drawn if there were several studies with nonsignificant findings and meta-analyses did not produce unequivocal results.

### Meta-analyses

Combined effect estimates were calculated by means of averaged associations across samples, weighting each observed association by the study’s sample size [44]. Eligible studies for inclusion in the meta-analyses reported categorical exposure variables with the unexposed employees (or employees with the lowest exposure category) as reference category. We synthesized studies reporting both Odds ratios and Hazard ratios and computed means of average associations as approximations of Relative risk ratios. This approximation is valid if the incidence rate of a study outcome is rare. The most completely adjusted risk estimates from each study and their corresponding confidence intervals or standard errors were used to compute combined effect estimates. When applicable, we computed additional subgroup analysis of the most comparable studies, i.e., studies that used the same exposure instrument measures, e.g., the Job content questionnaire (JCQ).

We computed random-effects models which estimate the mean of a distribution of true effects. The random effects model is recommended when there is reason to assume that the true effect vary from one study to the next [44]. The Q statistic was computed to assess the heterogeneity of studies (*p* < 0.05 rejects the null hypothesis of homogeneity). The I^2^ statistic shows the heterogeneity in percentages. To address the potential problem of publication bias, we computed the fail-safe N statistic which indicates the number of studies reporting null results that would be required to reduce the overall effect to non-significant [45]. All of the computed statistics were carried out by the Comprehensive Meta-Analysis (version 2) software, Biostat, Englewood, USA [46].

## Results

### Identified studies

**Fig. 1:**
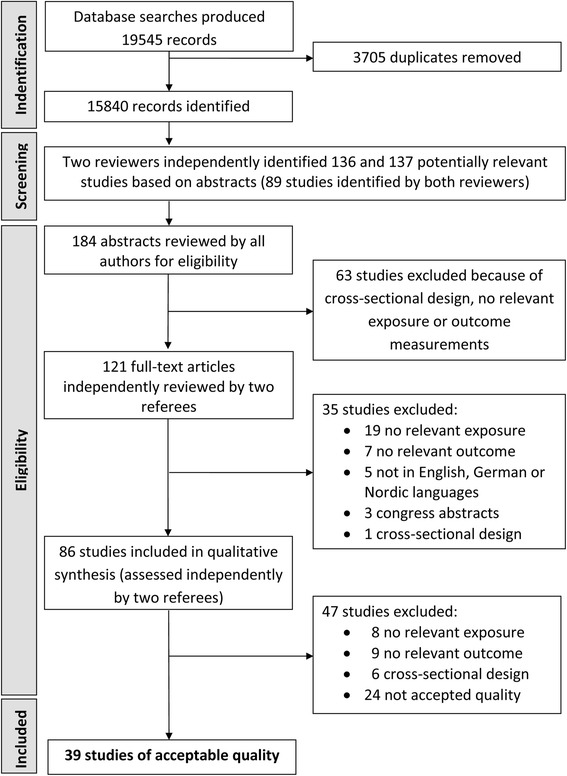
Flow chart for selection of studies

Of the 19545 abstracts, we identified 39 studies that fulfilled the inclusion criteria and satisfied the criteria for quality [12, 32, 43, 47–82]. Figure 1 depicts the identification, screening, eligibility, and inclusion processes. Studies excluded in the initial screening did not fulfill any of the inclusion criteria, or were duplicates (identified by Endnote reference library program, *n* = 3705). In all, 184 studies were considered as potentially relevant in the initial screening process. Of these 184 studies, 63 were excluded because of cross-sectional design or irrelevant outcome measures. In total, 121 [12, 24, 27–33, 43, 47–157] studies were independently reviewed in full text by two of the authors. Among these studies, 19 studies did not have a relevant exposure measure [33, 84, 90, 97, 105, 115–117, 119–121, 124, 131, 137, 141, 143, 147–149], seven studies did not report relevant outcome measures [87, 93, 103, 106, 110, 113, 129], five studies were not written in English, German, or a Nordic language [114, 125, 128, 136, 140], three studies were only reported as congress abstracts [92, 139, 144], and one study had a cross sectional design [130].

In total, 86 studies were included for assessment of quality [12, 24, 27–32, 43, 47–83, 85, 86, 88, 89, 91, 94–96, 98–102, 104, 107–109, 111, 112, 118, 122, 123, 126, 127, 132–135, 138, 142, 145, 146, 150–157]. Two referees independently assessed full-text articles from each study. Of these 86 studies, 23 were excluded because no relevant exposure full-filling the criteria was reported [27, 28, 95, 96, 146, 152, 154, 155], no relevant outcome was reported [24, 99, 109, 118, 127, 132, 138, 156, 157], and cross-sectional design [108, 126, 133, 134, 151, 153]. Of the remaining 63 studies [12, 29–32, 43, 47–75, 83, 85, 86, 88, 89, 91, 94, 98, 100–102, 104, 107, 111, 112, 122, 123, 135, 142, 145, 150], 24 studies with lower than 50% quality score [29–31, 83, 85, 86, 88, 89, 91, 94, 98, 100–102, 104, 107, 111, 112, 122, 135, 142, 145, 150] [123]: eleven did not report work-related risk estimates [29–31, 85, 88, 89, 91, 94, 111, 122, 142], twelve studies reported crude estimates only [83, 86, 98, 100–102, 104, 107, 112, 123, 135, 145, 150].

The table C of Additional file 1 presents characteristics and findings of the studies that did not meet the criterion quality score. The table D of Additional file 1 shows the scores of internal validity for accepted studies. The table E of Additional file 1 depicts the scores of internal validity for studies that were excluded by quality criteria.

### Overview of included studies

Of the 39 studies finally included, 19 studies were of high quality (internal validity of 66% or more) [12, 32, 43, 47–55, 76–82]. The highest score was 81% [43]. Table 1 presents a detailed overview of these 39 studies.

**Table 1:** Characteristics and results of the studies found to meet criteria for adequate quality

Authors, year publication, Country (subjects studied)	Exposures investigated (instruments used)	Employee groups (types of work), number of subjects	Outcomes (definition of disability), number of cases	Summary (with OR, RR, HR)	Control for confounders
Ahola et al., 2011, Finland [48]	Weekly hours; job strain (JCQ) (no test of control and demands); team climate (HOQ); job insecurity	General working population, *n* = 3164	Disability pension awards, registry, *n* = 208	Weekly hours (>40): OR = 0.78 (0.46–1.31) High job strain: **OR = 1.78 (1.10–2.87)** Poor team climate: OR = 1.25 (0.87–1.79) Job insecurity: OR = 1.23 (0.90–1.69)	Sex, age, mental and physical health, marital status, occupational grade, work-related factors, health behavior
Appelberg et al., 1996, Finland [12]	Interpersonal conflict at work	General working population, *n* = 15,348	Disability pension awards, registry, *n* = 364	Women: interpersonal conflict: **RR = 1.56 (1.01–2.39)** Men: interpersonal conflict: RR = 1.15 (0.74–1.81)	Sex, age, social class, general health status
Blekesaune et al., 2005, Norway [75]	Job stress (two single items); Decision authority (two single items) (job exposure matrix, no validated instruments)	General working population, *n* = 19,114	Disability pension awards, registry, *n* = 1257	*Men* Job stress: logit = 0.08 (*p* > 0.05) Low autonomy: **logit = 0.11** (*p* < 0.05) *Women* Job stress: logit = −0.05 (*p* > 0.05) Low autonomy: logit = −0.08 (*p* > 0.05)	Age, sex, marital status, income, physical job strain
Brage et al., 2007, Norway [61]	Organizational job stress; psychological job stress	General working population, *n* = 1152	Low back disability, defined as long term sickness absence (>56 days), rehabilitation allowance or disability awards, registry, *n* = 131	Organizational job stress: RR = 1.18 (0.97–1.43) Psychological job stress: RR = 1.17 (0.97–1.43)	Sex, age, marital status, education, life-style, psychosocial factors, emotional distress, work-related factors
Canivet et al., 2012, Sweden [54]	Job demands (JCQ), decision latitude (JCQ), job strain (JCQ), job support (Swedish version of JCQ)	Middle-aged general working population, *n* = 3359 women & 3181 men	Disability pension awards, registry, *n* = 672 women & 477men	Men High strain: **HR = 1.9 (1.4–2.7)** High demands: **HR = 1.7 (1.3–2.2)** Low decision latitude**: HR = 1.4 (1.1–1.9)** Low support: **HR = 1.3 (1.1–1.6)** Women High strain: **HR = 2.1 (1.6–2.7)** High demands: **HR = 1.8 (1.5–2.2)** Low decision latitude**: HR = 1.4 (1.1–1.8)** Low support: **HR = 1.2 (1.03–1.4)**	Sex, age, health status, occupational class, marital status, economical situation, alcohol consumption, smoking, BMI
Christensen et al., 2008, Denmark [70]	Decision authority (single item); information; variation in work	General working population, *n* = 8298	Disability pension awards, registry, *n* = 447	Men: Low decision authority: **HR = 1.82 (1.26–2.63)** Low variation in work: **HR = 1.75 (1.21–2.53)** Low information: HR = 0.96 (0.65–1.43) Women: Low decision authority: **HR = 1.41 (1.02–1.96)** Low variation in work**: HR = 1.40 (1.06–1.84)** Low information: RR = 1.34 (0.95–1.89)	Age, sex, smoking, BMI, ergonomic work environment exposures
Clausen et al., 2014, Denmark [76]	Influence at work (four items), Quality of leadership (four items), Work pace (single item), Quantitative demands (single item) All items from COPSOQ	General working population, *n* = 40,554	Disability pension awards, registry, *n* = 929	Influence at work (ref = high) Medium HR = 0.95 (0.85–1.13) Low **HR = 1.28 (1.05–1.55)** Quality of leadership (ref = high) Medium HR = 0.87 (0.73–1.03) Low HR = 0.91 (0.76–1.11) Work pace (ref = low) Medium HR = 0.90 (0.71–1.14) High HR = 1.06 (0.84–1.34) Quantitative demands (ref = low) Medium **HR = 0.82 (0.69–0.97)** High HR = 0.93 (0.77–1.12) Interaction effects Influence at work*Quantitative demands *p* = 0.65 Influence at work*Work pace *p* = 0.69 Quality of leadership*Quantitative demands ***p*** **< 0.01** Quality of leadership *Work pace ***p*** **< 0.01**	Age, sex, smoking, occupational group, psychosocial work conditions
Claussen et al., 2009, Norway [56]	Job control (decision authority); shift work	General working population, *n* = 9195	Disability pension awards, registry, *n* = 498	Job control: **OR = 2.07 (1.71–2.71)** Shift work: OR = 1.07 (0.71–1.57) Job strain: OR = 1.17 (0.96–1.42)	Sex, age, income, occupation, general health, mental distress
Claussen et al., 2009, Norway [57]	Job control (decision authority); shift work	General working population, *n* = 9195	Disability pension awards, registry, *n* = 498	Job control: **OR = 1.82 (1.35–2.44)** Shift work: OR = 1.07 (0.71–1.61) Job strain: OR = 1.17 (0.96–1.42)	Sex, age, ethnic group, income, occupation, general health, mental distress
Falkstedt et al., 2014, Sweden [77]	Job control (job exposure matrix–decision authority/skill discretion0)	Cohort of all Swedish children born in 1948 and 1953, men *n* = 7920/women *n* = 7191	Disability pension awards, registry, men *n* = 811/women *n* = 1372	Men Job control (ref: high) Medium-High **HR = 1.64 (1.35–1.98)** Medium-Low **HR = 2.01 (1.64–2.47)** Low **HR = 2.26 1.87–2.73)** Women Job control (ref: high) Medium-High **HR = 1.27 (1.02–1.55)** Medium-Low **HR = 1.26 (1.02–1.58)** Low **HR = 1.85 (1.50–2.28)**	Sex, age
Friis et al., 2008, Denmark [52]	Working schedule; work speed/pressure; busy at work; influence at work (single item)	All Danish nurses, *n* = 12,028	Disability pension awards, registry, *n* = 689	Working schedule (ref = day) Evening work: **HR = 1.51 (1.21–1.89)** Night work: **HR = 1.45 (1.11–1.91)** Shift work: HR = 0.94 (0.73–1.20) Busy at work (ref = almost always) Often/sometimes: HR = 0.91 (0.70–1.19) Not often/never: HR = 0.86 (0.63–1.18) Work speed/pressure: HR = 1.10 (0.94–1.29) Influence at work (major): **HR = 1.39 (1.17–1.67)**	Sex, age, working area, psychical demands, leisure time physical activity, BMI, alcohol, smoking, marital status and spouse’s income, income, place of residence
Hagen et al., 2002, Norway [58]	Excessive job demands, authority to plan own work, concentration and attention	General working population, *n* = 34,754	Disability pension awards due to back pain, registry, *n* = 715	None significant results in the fully adjusted model (fully adjusted estimates not shown)	Sex, age, occupational factors, general health status, health behavior, psychological and social variables
Hagen et al., 2006, Norway [74]	Concentration and attention; Stress and tension; Authority to plan own work (single item)	General working population, *n* = 38,426	Disability pension awards due to back disease, registry, *n* = 4146	Concentration and attention (ref = almost never) Fairly infrequently HR = 1.2 (0.8–1.8) Fairly often **HR = 1.6 (1.1–2.4)** Nearly always **HR = 2.6 (1.6–4.1)** Stress and tension (ref = not at all) Not much **HR = 0.7 (0.5–0.9)** A good deal HR = 0.8 (0.6–1.0) Almost all the time HR = 0.9 (0.6–1.3) Authority to plan own work (ref = almost all the time) A good deal HR = 0.9 (0.7–1.1) Not much HR = 1.1 (0.9–1.5) Not at all **HR = 1.5 (1.3–2.1)**	Sex, age
Hinkka et al., 2013, Finland [59]	Shift work, work control (single item; decision authority), opportunities for mental growth, support from supervisor, feedback from supervisor	Random sample of civil service employees, *n* = 937	Disability pension awards, registry, *n* = 44	Shift work (ref = daytime) Shift or period work: **HR = 3.64 (1.66–7.99)** Part-time work: HR = 2.29 (0.48–10.95) Work control (ref = low) Moderate: HR = 0.68 (0.32–1.45) High: **HR = 0.25 (0.07–0.87)** Mental growth (ref = rather or very little) Somewhat: HR = 0.89 (0.41–1.93) Rather or very much: HR = 1.07 (0.43–2.68) Support (ref = almost never or seldom) Sometimes HR = 0.99 (0.47–2.11) Rather or very often: HR = 0.39 (0.15–1.02) Feedback (ref = almost never or seldom) Sometimes HR = 0.87 (0.40–1.89) Rather or very often: HR = 1.28 (0.44–3.73)	Sex, age, education, occupational class, physical work exposure, physical activity
Holmberg et al., 2006, Sweden [66]	Job demands (JCQ); decision latitude (JCQ)	Rural male farmers and non-farmers, *n* = 1347 (cases = 39)	Disability pension awards, registry, *n* = 39	None significant results in the fully adjusted model (fully adjusted estimates not shown)	Sex, age, occupation (farmer/non-farmer), psychical work load, education, BMI, tobacco, alcohol, psychiatric symptoms, specific low back symptoms
Hublin et al., 2010, Finland [32]	Shift work	General working population, *n* = 20,142	Disability pension awards, registry, *n* = 857	Shift work (ref = daytime) All men: Night time T1 or T2: RR = 0.51 (0.17–1.49) Shift work T1: RR = 1.12 (0.70–1.78) Shift work T2: RR = 1.13 (0.67–1.90) Shift work T1 and T2: RR = 0.70 (0.48–1.03) All women: Night time T1 or T2: RR = 0.94 (0.35–2.51) Shift work T1: RR = 0.93 (0.48–1.82) Shift work T2: RR = 1.32 (0.72–2.40) Shift work T1 and T2: RR = 0.79 (0.43–1.43)	Sex, age, marital status, social class, education, smoking, binge drinking/alcohol consumption, hypertension, BMI, physical activity, work-related factors, general health factors, life satisfaction
Jensen et al., 2012, Denmark [55]	Decision latitude (JCQ), demands (JCQ, the Danish version), shift work	Gainfully employed nurses’ aides, *n* = 3332	Disability pension awards, registry, *n* = 540 (Additionally: voluntary early retirement)	Decision latitude (ref = high): HR = 0.90 (0.72–1.12) Demands (ref = low): HR = 0.92 (0.75–1.13) Shift work (ref = mainly day work) Mainly evening work: **HR = 1.29 (1.03–1.60)** Mainly night work: HR = 1.18 (0.87–1.61) Mixed: HR = 0.97 (0.74–1.27)	Sex, age, marital status, health status, BMI, smoking, physical activity, education, mechanical work load
Juvani et al., 2014, Finland [78]	Effort-reward imbalance (one item on effort and three items on rewards, adapted from the standard 10 item ERI scale developed by J. Siegrist)	Cohort of public-sector employees, *n* = 51,874	Disability pension awards due to all diagnosis (*n* = 4542), depression (*n* = 890), musculoskeletal diagnoses (*n* = 2001), ischemic heart disease (*n* = 87), registry	Work unit-level ERI All diagnosis 4^th^ highest HR = 1.07 (0.97–1.18) Depression 4^th^ highest **HR = 1.63 (1.31–2.04)** Musculoskeletal 4^th^ highest HR = 1.02 (0.88–1.99) Ischemic heart disease 4^th^ highest HR = 0.95 (0.48–1.88) Individual-level ERI All diagnosis 4^th^ highest **HR = 133 (1.20–1.47)** Depression 4^th^ highest **HR = 1.90 (1.51–2.40)** Musculoskeletal 4^th^ highest **HR = 1.32 (1.13–1.53)** Ischemic heart disease 4^th^ highest HR = 0.93 (0.47–1.84)	Sex, age, place of residence, socioeconomic status, baseline health and work-related characteristics (Job strain)
Karkkainen et al., 2013, Finland [65]	Work-time schedules (day, night, evening, shift work)	General working population (Finnish twins), *n* = 16,028	Disability pension awards due to musculoskeletal diagnoses, registry, *n* = 1297	Work type (ref = day work) Night: HR = 0.74 (0.17–3.18) Evening: **HR = 3.61 (1.21–10.76)** Shift: HR = 1.17 (0.83–1.65) (NB: insignificant results for within twin pairs)	Sex, age, BMI, marital status, physical work exposure, education, occupational class, physical activity
Krause et al., 1997, Finland [62]	Weekly work hours; Overwork index; Shift work; Deadlines (ref = weekly or less, daily or more often)	General male working population, *n* = 968	Disability pension awards, questionnaire, *n* = 67	Weekly work hours (ref= > 40) 40–44: OR = 1.26 (*p* > 0.05) 45–59: OR = 1.49 (*p* > 0.05) **> = 60: OR = 2.49 (** ***p*** **< 0.05)** Overwork: OR = 1.31 (*p* > 0.05) Shift work (ref = no shift) Regular shift: **OR = 0.24 (** ***p*** **< 0.05)** Irregular shift: OR = 0.98 (*p* > 0.05) Deadlines (ref = weekly or less) Daily or more: OR = 1.22 (*p* > 0.05)	Sex, age, economic factors, health status, health behavior
Krokstad et al., 2002, Norway [50]	Job control (decision authority); high demands in concentration and attention	General working population, *n* = 62,369	Disability pension awards, registry, *n* = 4750	Men (20–49 y) Job control (ref = high): **HR = 1.40 (1.00–1.94)** Men (50–66 y) Job control (ref = high): **HR = 1.34 (1.13–1.59)** Psych. demands (ref = low): HR = 1.11 (0.99–1.25) Women (20–49 y) Job control (ref = high): **HR = 1.29 (1.04–1.60)** Women (50–66 y) Job control (ref = high): **HR = 1.31 (1.09–1.59)** Psych. demands (ref = low): **HR = 1.33 (1.13–1.57)**	Sex, age, educational level, employment status, health status, psychosocial risk factors, health behaviour
Labriola et al., 2007, Denmark [71]	Decision authority (ref = high); skill discretion (ref = high); social support (ref = high); conflicts (ref = low); psychological demands (ref = low) (JCQ)	General working population, *n* = 4177 (cases = 140)	Disability pension awards, registry, *n* = 140	Decision authority: OR = 1.14 (0.77–1.71) Skill discretion: OR = 1.24 (0.82–1.86) Social support: OR = 0.73 (0.48–1.12) Conflicts: OR = 1.20 (0.78–1.84) Psychological demands: OR = 0.90 (0.59–1.38)	Sick leave, gender, age, socioeconomic position, smoking, BMI, ergonomic work-related factors
Lahelma et al., 2012, Finland [60]	Work arrangements (shift work; temporary work contract; working overtime (hours > 40 per week)), decision latitude (JCQ), job demands (JCQ), social support (Sarason)	Employees of the city of Helsinki, *n* = 6525	Disability pension awards, registry, *n* = 525	*All-cause disability* Men Shift work (ref = no): HR = 1.16 (0.70–1.93) Temporary work (ref = no): HR = 0.84 (0.36–1.96) Overtime (ref = no): HR = 0.95 (0.56–1.60) Low decision latitude: HR = 1.41 (0.90–2.21) High job demands: HR = 0.75 (0.40–1.39) Social support: HR = 0.88 (0.57–1.37) Women Shift work (ref = no): HR = 1.02 (0.79–1.31) Temporary work (ref = no): HR = 1.03 (0.75–1.42) Overtime (ref = no): HR = 1.02 (0.75–1.39) Low decision latitude: **HR = 1.34 (1.07–1.68)** High job demands: HR = 1.13 (0.88–1.44) Social support: HR = 0.92 (0.74–1.15) *Musculoskeletal diseases* Shift work (ref = no): HR = 1.04 (0.75–1.44) Temporary work (ref = no): HR = 0.70 (0.40–1.20) Overtime (ref = no): HR = 0.85 (0.53–1.36) Low job control: **HR = 1.44 (1.07–1.93)** High job demands: HR = 0.94 (0.65–1.34) Social support: HR = 0.80 (0.58–1.11) *Mental disorders* Shift work (ref = no): HR = 1.03 (0.66–1.60) Temporary work (ref = no): HR = 1.06 (0.61–1.81) Overtime (ref = no): HR = 1.12 (0.71–1.78) Low job control: **HR = 1.67 (1.12–2.49)** High job demands: HR = 1.48 (1.00–2.18) Social support: HR = 0.82 (0.56–1.19)	Sex, age, occupational class, physical job exposure
Laine et al., 2009, Finland [67]	Job strain (JCQ), job control, job demands (JCQ)	Finish public sector employees, *n* = 25,150	Retired because of work disability, questionnaire, *n* = 93	*Job strain* (ref = low) active: OR = 1.69 (0.62–2.87) Passive: **OR = 2.82 (1.34–5.96)** high: **OR = 2.60 (1.26–5.34)** *Job control* (ref high) low control: **OR = 2.09 (1.02–4.30)** *Job demands* (ref = low) High demands: OR = 1.76 (0.94–3.30)	Sex, age, socioeconomic position, smoking, alcohol, physical activity, obesity
Lund et al., 2001, Denmark [68]	Job demands, Decision authority; Social support; Skill discretion (JCQ, 20 items)	Waste collectors (2412) and municipal workers (1460) in Denmark. *N* = 2.618 male workers	Disability pension, or sick leave benefits >2 months, self reported, *n* = 67	Skill discretion (ref = high quartile) Mid 2 quartile: OR = 1.68 (0.74–3.82) Mid1 quartile: OR = 1.25 (0.49–3.10) Low quartile: **OR = 2.70 (1.10–6.70)** Insignificant results for Job demands, Decision authority and Social support (estimate not reported).	Sex, age, mechanical exposure, asthma, chronic bronchitis, musculoskeletal disorders, smoking status, Body Mass Index (BMI), alcohol consumption, marital status
Lund et al., 2003, Denmark [63]	Decision authority; job demands; social support; conflict at work; employee development; supplementary training (JCQ: 18 items)	General working population, *n* = 3318	Disability pension awards, or sick leave benefits >10 weeks, registry, *n* = 77	Decision authority: *p* > 0.05 Job demands: *p* > 0.05 Social support: *p* > 0.05 Conflict at work: *p* > 0.05 *Employee development* (ref = high) Medium: OR = 1.2 (0.62–2.14) **Low: OR = 2.2 (1.11–4.39)** *Supplementary training* (ref = high) Medium: OR = 1.7 (0.77–3.90) **Low: OR = 2.4 (1.11–5.26)**	Sex, age, marital status, health, BMI, smoking, company size, public or private sector
Mantyniemi et al., 2012, Finland [47]	Job strain based on work unit and occupational title, respectively (derived from JCQ; conflicting demands not included) (no test of demands and control)	Public sector employees, *n* = 69,842	Disability pension awards, registry, *n* = 2572	Men All cause disability: work unit based **HR = 1.28 (1.07–1.53)**/occupation based **HR = 1.40 (1.15–1.71)** Musculoskeletal diseases: work unit based **HR = 1.66 (1.26–2.20)**/occupation based **HR = 2.41 (1.81–3.21)** Depression: work unit based **HR = 1.59 (1.03–2.47)**/occupation based HR = 1.30 (0.78–2.16) Coronary heart disease: work unit based **HR = 2.14 (1.01–4.50)**/occupation based **HR = 2.37 (1.10–5.10)** Women All cause disability: work unit based HR = 1.07 (0.98–1.17)/occupation based **HR = 1.17 (1.04–1.31)** Musculoskeletal diseases: work unit based **HR = 1.48 (1.31–1.67)**/occupation based **HR = 2.21 (1.91–2.57)** Depression: work unit based HR = 1.15 (0.97–1.37)/occupation based **HR = 1.24 (1.00–1.53)** Coronary heart disease: work unit based HR = 1.18 (0.57–2.45)/occupation based HR = 0.98 (0.39–2.47)	Sex, age, health status, socioeconomic position
Robroek et al., 2013, European countries [73]	High time pressure (single item); low decision latitude (JCQ: two items, one on authority and one on skill discretion); low rewards (JCQ)	General working population >49 y, *n* = 4923	Disability pension awards, questionnaire, *n* = 6.2 per 1000 person-years	High time pressure HR = 1.14 (0.77–1.70) Low decision latitude : **HR = 1.77 (1.10–2.84)** Low rewards HR = 1.44 (0.96–2.16)	Sex, age, health status, physical work exposure, education, marital status, BMI, smoking
Ropponen et al., 2012, Finland [72]	Work-time schedules (day, shift, evening/night)	General working population (twins), *n* = 16,028	Disability pension awards due to Low Back Diagnoses, registry, *n* = 470	Men Day work = ref Shift work: HR = 1.36 (0.98–1.88) Evening/night work: HR = 0.62 (0.08–4.48) Women Day work = ref Shift work: HR = 0.92 (0.59–1.43) Evening/night work: HR = 0.1.52 (0.62–3.75)	Sex, age, Education, physical work exposure, physical activity, marital staus, BMI, smoking
Ropponen et al., 2013, Sweden [79]	Job demands (5 items); job control (7 items); social support at work (4 items); job strain	Cohort of all Swedish twins born between 1928 and 1958, *n* = 42,715	Disability pension awards due to musculoskeletal diagnoses, registry, *n* = 1774	Job demands (range 1–10; high score is low) **HR = 1.10 (1.02–1.20)** Job control (range 1–10; high score is high) **HR 0.93 (0.89–0.97)** Social support (range 1–10; high score is high) **HR = 1.08 (0.98–1.19)** Job strain (ref = low) High strain HR = 0.93 (0.73–1.19) Active HR = 0.89 (0.75–1.07) Passive **HR = 1.25 (1.07–1.46)** ISO-strain **HR = 1.27 (1.04–1.57)**	Sex, age, education, married, children working at home
Samuelsson et al., 2013, Sweden [49]	Job demands (continuous); job control (continuous); Social support (continuous); job strain (validated JEM, based on principal component factor analysis of the Swedish questionnaire items relating to work)	All twins born 1925–1958 in Sweden, *n* = 59,893	Disability pension awards with mental diagnoses, registry, *n* = 7709	Whole cohort Low job demands: HR = 1.07 (0.98–1.15) High job control**: HR = 0.93 (0.89–0.97)** High social support: HR = 1.12 (1.01–1.24) Job strain (ref = low strain) High: HR = 0.96 (0.75–1.22) Active: HR = 0.97 (0.81–1.16) Passive: HR = 1.26 (1.05–1.50) Iso-strain: HR = 1.41 (1.12–1.77) Twin cohort Low job demands: HR = 1.23 (1.06–1.43) High job control: **HR = 0.91 (0.83–0.99)** High social support: HR = 1.00 (0.79–1.23) Job strain (ref = low strain) High: HR = 0.65 (0.41–1.03) Active: HR = 0.78 (0.56–1.08) Passive: HR = 1.04 (0.76–1.44) Iso-strain: HR = 1.09 (0.76–1.44)	Sex, age, marital status, education, occupational class
Sinokki et.al., 2010, Finland [53]	Social support at work, supervisor and coworkers (JCQ)	General working population, *n* = 3414	Disability pension awards, registry, *n* = 257	Support from supervisor (ref. = high) Low: **OR = 1.70 (1.21–2.38)** Support from co-workers (ref. = high) Low: OR = 1.35 (0.86–2.14)	Sex, age, marital status, occupational grade, physical activity, BMI, alcohol consumption, smoking, and either physical illness, mental disorders, sleeping difficulties
Sterud T. al., 2013, Norway [80]	Job demands (single item), Job control (three items), supportive leadership (three items), bullying/harassment (three items), monotonous work (single item) Some items have been tested for psychometric quality	Representative cohort from the general working population, *n* = 6745	Disability pension awards, questionnaire, *n* = 176	Job demands OR = 0.82 (0.53–1.26) Job control OR = 0.84 (0.54–1.30) Supportive leadership **OR = 1.61 (1.02–2.56)** Bulling/harassment OR = 1.52 (0.78–2.93) Monotonous work **OR = 1.53 (1.09–2.16)**	Sex, age, psychological distress, mechanical factors at work
Støver et al., 2013, Norway [81]	Cumulative summation index of 11 psychosocial work exposure questions	Cohort of all individuals aged 40–42 years in 1988–1989, living in Nordland county, Norway, *n* = 5749	Disability pension awards, registry, *n* = 1944	Five point increase on the cumulative index for psychosocial work environment **HR = 1.22 (1.04–1.44)**	Sex, age, baseline health, smoking, alcohol consumption, education, physical-, chemical- and mechanical work exposures
Thielen et al., 2013, Denmark [82]	Amount of work (three items, COPSOQ)	General working population, *n* = 5758	Disability pension awards, registry, *n* = 2201	Amount of work (ref = low) Middle HR = 0.95 (0.68–1.31) High HR = 0.67 (0.44–1.01) Mental demands/depression (ref = low/low) High/low HR = 0.66 (0.41–1.05) Low/High **HR = 2.45 (1.71–3.52)** High/High **HR = 2.08 (1.26–3.40)**	Sex, age, marital status, education, alcohol consumption, smoking, obesity, muscular-skeletal pain, depressive symptoms
Tüchsen et al., 2008, Denmark [64]	Shift workers compared with permanent day workers	General working population sample, *n* = 3980 women and 4025 men	Disability pension, registry, *n* = 253 women and 173 men	Women Shift work **HR = 1.34 (1.02–1.75)** Men Shift work HR = 1.18 (0.96–1.46)	Sex, age, general health, SES, BMI, smoking status and ergonomic exposure
Vahtera et al., 2005, Finland [43]	Downsizing (ref = reductions in personnel less than 8% vs. minor downsizing (8–18%) and major downsizing (more than 18%).	Municipal employees, *n* = 5043 men and 14,239 women	Disability pension awards, registry, *n* = 68 men and 155 women	Men All cause disability: **HR = 1.46 (1.02–2.08)** Psychiatric diseases: HR = 1.26 (0.70–2.26 Musculoskeletal diseases: HR = 1.29 (0.65–2.59) Other causes: **HR = 1.55 (1.10–2.19)** Women All cause disability: **HR = 1.81 (1.22–2.70)** Psychiatric diseases: HR = 1.26 (0.63–2.54) Musculoskeletal diseases: HR = 1.81 (0.94–3.90) Other causes: **HR = 1.89 (1.27–2.81)**	Sex, age, occupational status, type of employment contract, and geographical area
Vahtera, J. et al., 2010, Finland [51]	Self-assessed worktime control (7 items scale); Co-worker assessed worktime control (7 items scale) (Validated instrument)	Public sector employees, *n* = 30,700	Disability pension awards, registry, *n* = 1178	Self-assessed worktime control All**: HR = 0.87 (0.80 to 0.94)** Men**: HR = 0.97 (0.82 to 1.15)** Women**: HR = 0.86 (0.78 to 0.94**) Co-worker assessed worktime control All**: HR = 0.76 (0.67 to 0.87)** Men**: HR = 0.94 (0.72 to 1.23)** Women: **HR = 0.71 (0.61 to 0.83)**	Sex, age, socioeconomic status, work-related factors, health risk behavior, health indicators
Virtanen et.al., 2010 UK [69]	Organizational change	Civil servants (office staff), *n* = 4682	Retirement due to health reasons or left The Civil Service and subsequently classified themselves as long term sick, *n* = 239	Organizational change (Yes vs. not planned) **HR = 1.61 (1.23–2.10)**	Sex, age, duration of employment, grad, perceived health, marital status, material problems, housing tenure, car access, long term illness, general health questionnaire

Fourteen studies were based on a Finnish population [12, 32, 43, 47, 48, 51, 53, 59, 60, 62, 65, 67, 72, 78], nine studies on a Norwegian population [50, 56–58, 61, 74, 75, 80, 81], nine studies on a Danish population [52, 55, 63, 64, 68, 70, 71, 76, 82], five studies on a Swedish population [49, 54, 66, 77, 79]. One study was based on a population from the UK [69], and one study used data from several European countries [73]. Twenty-five studies were based on general working population samples (ns range: 3164 – 69842), five studies on public-sector employees, three studies on municipal employees, four studies on specific occupations, and two studies based on twins. Thirty-three studies identified disability retirement by registries while six studies depended on self-report questionnaire.

Studies reporting several work factors are presented in two or more sections in this article.

### Overall appraisal of the work environment

One representative general-population study by Støver and coworkers [81] found that overall poor psychosocial work environment predicted disability retirement, based on the number of “poor psychosocial work exposures” out of 11 questions encompassing variation, feedback, support, influence (control), demands, reorganization, and bullying.

### Studies of psychological factors

#### Job demands

Twenty studies addressed aspects of job demands [49, 50, 52, 54, 55, 58, 60, 62, 63, 66–68, 71, 73–76, 79, 80, 82] (Table 1). The study populations were: all Danish nurses [52], nurses’ aides [55], male waste collectors & municipal employees [68], rural male farmers and non-farmers [66], Finnish public sector employees [67], employees of the city of Helsinki [60], and in fourteen studies data from the general working population [49, 50, 54, 58, 62, 63, 71, 73–76, 79, 80, 82]. Except for five studies [62, 67, 68, 73, 80], all used registry data to assess disability retirement.

Ten studies assessed job demands with measures from, or derived from the Job Content Questionnaire (JCQ) [49, 54, 55, 60, 63, 66–68, 71, 79], whereas one study assessed “amount of work” with the Copenhagen Psychosocial Questionnaire (COPSOQ) [82]. In Eight of the remaining nine studies, aspects of job demands were assessed with single item measures [50, 52, 58, 62, 73, 74, 76, 80]. Finally, one study assessed aspects of job demands with a non-validated two item scale [75].

Aspects of job demands were reported to predict subsequent disability retirement in four out of total twenty studies. Job demands were positively related to subsequent disability retirement in two [54, 79] of the eleven studies that measured demands by validated instruments, one of which pertained to retirement due to mental diagnoses [48]. However Ropponen et al. [79] reported that *low demands* was associated with an increased risk of disability awards with musculoskeletal diagnoses and Clausen et al. [76] found that medium-level of demands reduced risk of disability pension awards compared to low demands.

Specific aspects of job demands were significantly related to subsequent disability retirement in two of the eight studies that were based on single item demand measures [50, 74]. Krokstad et al [50] found significant associations between high levels of “*concentration and attention*” and subsequent disability retirement among women in the general working population, but not among men. Based on the same population and measurements as Krokstad et al, Hagen et al [74] found a significant sex and age-adjusted increased risk.

**Fig. 2:**
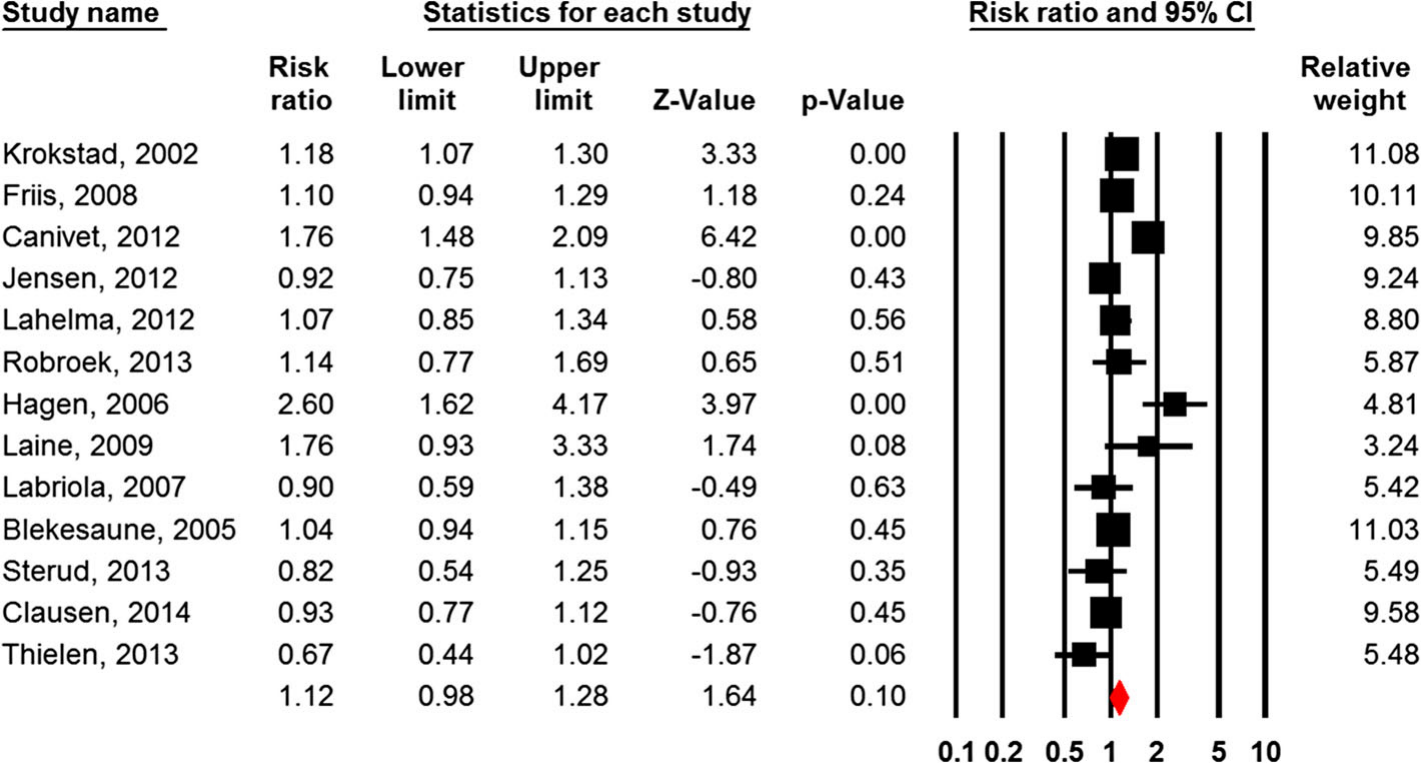
Forest plot Job Demands

Thirteen of the twenty studies were suitable for meta-analysis [50, 52, 54, 55, 60, 67, 71, 73–75, 80–82]. Figure 2 shows the forest plot and the results from the meta-analysis. The combined relative risk estimate of the thirteen studies did not show an association (RR = 1.12; 95% CI = 0.98-1.28). The Q statistics showed substantially heterogeneity between studies (Table 2). The reason may be that the different studies measured different exposures (e.g., job stress, busy at work, high time pressure, demands in concentration and attention, JCQ-job demands). In addition, some studies measured the outcome by questionnaire whereas other studies reported registry data. The subgroup analysis of the four comparable studies which measured job demands with JCQ and based information on disability pension on registries [54, 55, 60, 71], showed no significant association (RR = 1.14; 95% CI = 0.80-1.62). The heterogeneity between these studies was substantial (*p* < 0.01).

**Table 2:** Meta-analysis of the effects of work-related factors on disability pension retirement (random effects model)

Factors	N studies	Combined effect estimates		Q	I^2^	NMS
		RR	95% CI			
Control	16	1.40	1.21-1.61	131.1*	88.6	623*
Control (sub group)	5	1.33	1.04-1.69	14.5*	72.5	21*
Demands	13	1.12	0.98-1.28	60.6*	80.2	-
Demands (sub group)	4	1.14	0.80-1.62	27.8*	89.2	-
Strain	5	1.45	0.96-2.19	35.6*	88.8	-
Support	3	1.17	0.86-1.61	8.9*	77.4	
Shift work (all types)	8	1.08	0.92-1.28	19.1*	63.3	-

Note: *p < .001

*NMS* Number of missing studies that would bring *p* value to > 0.05, based on the fail-safe N method

Stratified analyses for studies with single item (*n* = 7) and multiple item (*n* = 6) measurements of demands showed a RR of 1.08 (95% CI: 0.90-1.30) for single item and an RR of 1.16 (95% CI: 0.92-1.47) for multiple item measures.

Stratified analyses for studies with high quality (*n* = 8) and acceptable quality (*n* = 5) did not reveal a difference in results of the effects of demands on disability pension (high quality studies RR = 1.06; 95% CI = 0.89-1.27 vs acceptable quality studies RR = 1.26; 95% CI = 0.96-1.65).

#### Repetitive work tasks (monotonous work)

One representative general-population study by Sterud [80] found that reporting having repetitive work tasks three quarters of the work day or more predicted disability pension. Repetitive work tasks was measured with a single item: Does your job consist of constantly repeated tasks, meaning that you do the same thing hour after hour?”

#### Control

The control dimension pertains to freedom to choose between alternatives. Twenty-four studies addressed aspects of job control [49–52, 54–60, 63, 66–68, 70, 71, 73–77, 79, 80]. The study populations were: all Danish nurses [52], nurses’ aides [55], employees of the city of Helsinki [60], Finnish civil service employees [59], rural male farmers and non-farmers [66], Finnish public sector employees [51, 67], male waste collectors and municipal workers [68], and in sixteen studies data from the general working population [49, 50, 54, 56–58, 63, 70, 71, 73–77, 79, 80]. In nineteen studies, disability retirement was extracted from registry data, whereas five studies were based on a questionnaire [62, 67, 68, 73, 80].

Twelve studies used measures from or derived from the JCQ [49, 54, 55, 60, 63, 66–68, 71, 73, 77, 79]. This instrument defines the control dimension as *job*-*decision latitude* consisting of two factors: *decision authority* and *skill discretion* (required skills for the job). Nine of the twelve studies reported *decision latitude* [49, 54, 55, 60, 66, 67, 73, 77, 79], whereas two studies assessed *decision authority* and *skill discretion* separately, [68, 71] and one study assessed *decision authority* only [63]. Of the remaining twelve studies, one study measured influence at work with a four item scale from COPSOQ [76], one study assessed work-time control by a validated instrument (self-reported and co-worker-reported) [51], one study assessed job control with a three item scale [80], while one study measured decision authority-like aspects of control with a non-validated two item scale [75], and eight studies measured decision-authority like aspects of job control with single items [50, 52, 56–59, 70, 74].

Aspects of job control were significantly associated with subsequent disability retirement in 18 [49–52, 54, 56, 57, 59, 60, 67, 68, 70, 73, 75] of 24 studies. Seven [49, 54, 60, 67, 73, 77, 79] of the nine studies that assessed *decision latitude* with measures from the JCQ found a significant association with subsequent disability retirement [49, 54, 55, 60, 66, 67, 73]. None of the three studies that assessed *decision authority* with measures from JCQ found significant associations [63, 68, 71]. One of the two studies [68, 71] that assessed *skill discretion* with measures from JCQ found increased risk [68].

Aspects of job control were significantly associated with disability retirement in three [51, 75, 76] of the four studies that used other psychometric scales than the JCQ [51, 75, 76, 80]. Aspects of job control were significantly associated with subsequent disability retirement in seven [50, 52, 56, 57, 59, 70, 74] of the eight studies that used single-item measures [50, 52, 56–59, 70, 74].

**Fig. 3:**
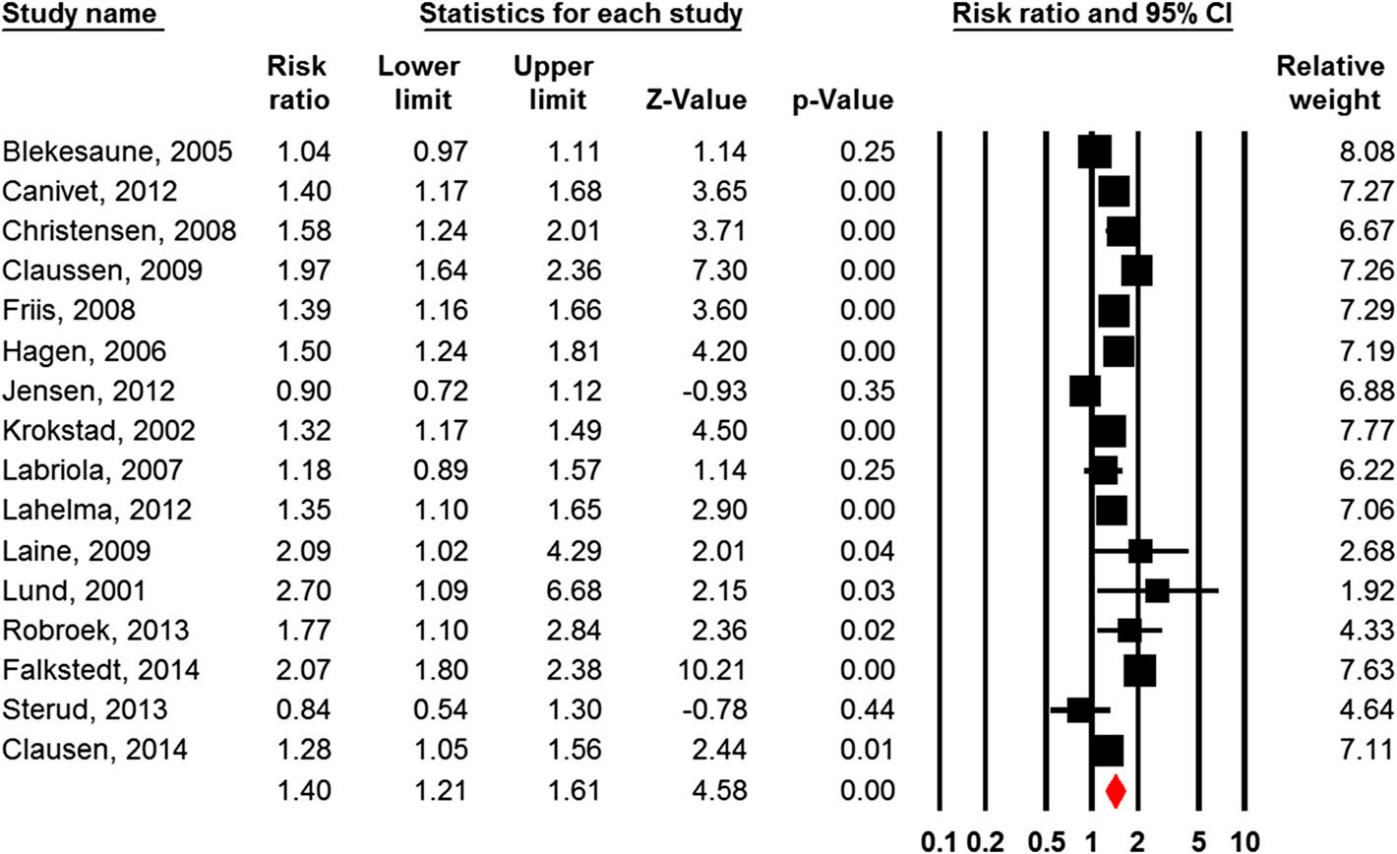
Forest plot Job Control

Sixteen of the 24 studies were suitable for meta-analysis [50, 52, 54–57, 60, 67, 68, 70, 71, 73, 75–77, 80]. The combined relative risk estimate of the six teen studies also showed a significant association (RR = 1.40; 95% CI = 1.21-1.61; Fig. 3). The fail-safe N estimates indicate that the observed effect estimates were robust (Table 2). Nevertheless, the Q-statistics indicated substantial heterogeneity between the included studies. A plausible reason is that different exposure and outcome measures have been investigated. The subgroup analysis between the most comparable studies [54, 55, 60, 67, 73], in which all studies measured decision latitude with the JCQ, showed a significant association (RR = 1.33; 95%CI = 1.04-1.69). Nevertheless, the Q-statistic (*p* < 0.01) showed substantial heterogeneity between these studies as well.

#### Job strain

Six studies addressed *job strain* [47–49, 54, 67, 79] referring to the combination of high level of demands and low level of control (the job–strain model; Karasek [3]) (Table 1). All of these assessed job strain with measures from or derived from the Job Content Questionnaire (JCQ) [5]. Mantyniemi et al [47] and Samuelsson et al [49] used aggregated scores (work unit/job title and Job Exposure Matrix, respectively) to determine strain. The studies by Ahola et al [48], Ropponen et al [79], Samuelsson et al [49] and Canivet et al [54] were based on data from the general working population, whereas the studies by Mantyniemi et et al [47] and Laine et al [67] pertained to Finnish public-sector employees. With the exception of the study by Ahola et al [48] that measured disability retirement by a questionnaire, all studies assessed disability retirement by registries.

Job strain was a significant predictor of subsequent disability retirement in four out of six studies [47, 48, 54, 67]. The studies with nonsignificant results for job strain showed an increased risk of disability for the combination of low job demands and low job control (passive jobs) [49, 79], hence control may be the decisive factor.

**Fig. 4:**
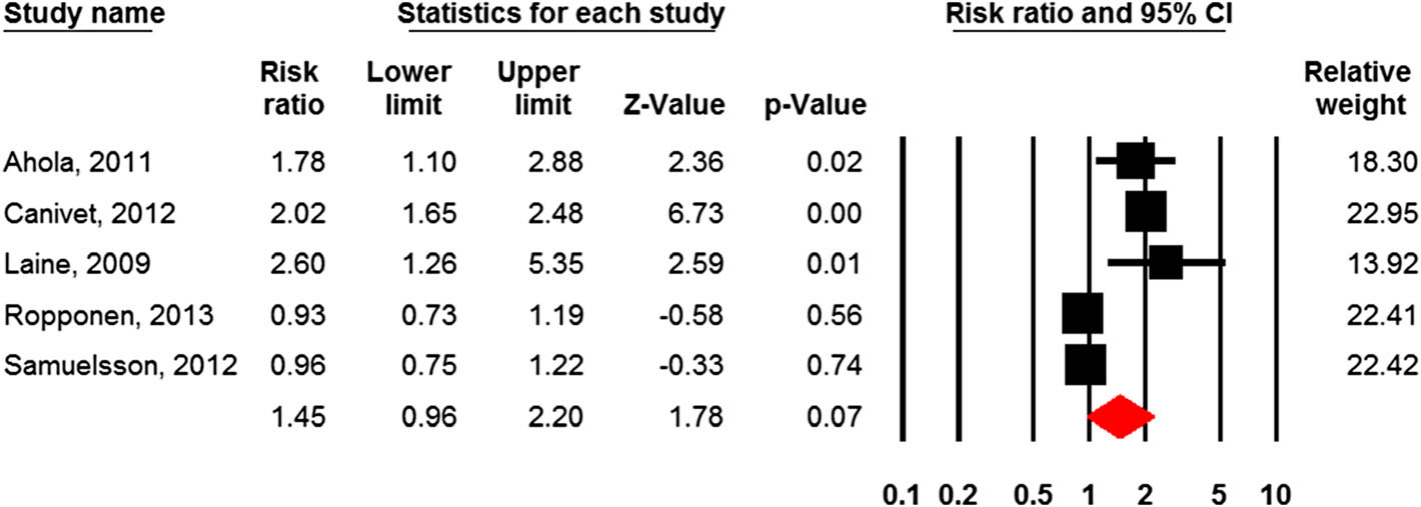
Forest plot Job strain

Of the six studies, five were suitable for meta-analysis [48, 49, 54, 67, 79]. Figure 4 and Table 2 shows that high job strain was borderline significantly associated with increased risk of disability pension (RR = 1.45; 95% CI = 0.96-2.19). The two studies with nonsignificant results for job strain were based on two samples drawn from the same population [48, 78], but were both included in this analysis. The Q-statistic indicates substantial heterogeneity between studies (*p* < 0.01).

#### Effort-reward imbalance (ERI)

High effort-reward imbalance and low rewards were found to predict disability pension due to depression, both in analyses of individual level and work-unit level of ERI scores in one study [78]. The population was Finnish public sector employees. Effort-reward imbalance was measured with a four-item questionnaire, one item measuring effort and three measuring rewards. Individual level scores of ERI also predicted disability retirement due to musculoskeletal disorders.

#### Development and training

One study of the Danish general working population study found significant effects of both low employee development and low supplementary training on the risk of registry based disability-pension awards [63].

### Studies of social factors

#### Social support

Social support refers to assistance, information, feedback, and emotional support. Nine studies addressed aspects of social support [49, 53, 54, 59, 60, 63, 68, 71, 79]. Data were extracted from the general working population in six of these studies [49, 53, 54, 63, 71, 79], whereas two studies were based on civil service employees [59] and employees of the city of Helsinki [60], respectively. In all of these studies, disability retirement was assessed by registry data. In the remaining study, the population was waste collectors and municipal workers, and disability retirement was assessed by questionnaire [68]. Seven studies assessed support with measures from the JCQ [49, 53, 54, 63, 68, 71, 79]. Of the remaining two studies, Hinkka et al [59] assessed support with a single item, whereas Lahelma et al [60] used another validated instrument.

Social support was related to subsequent disability retirement in three out of nine studies. Low supervisor support was found to increase the risk of subsequent disability retirement in the study by Sinokki et al [53] and Canivet et al [54]; whereas *high* social support was found to increase the risk in the study by Samuelsson et al [49]. In all these three studies, aspects of social support were assessed with measures from the JCQ.

**Fig. 5:**
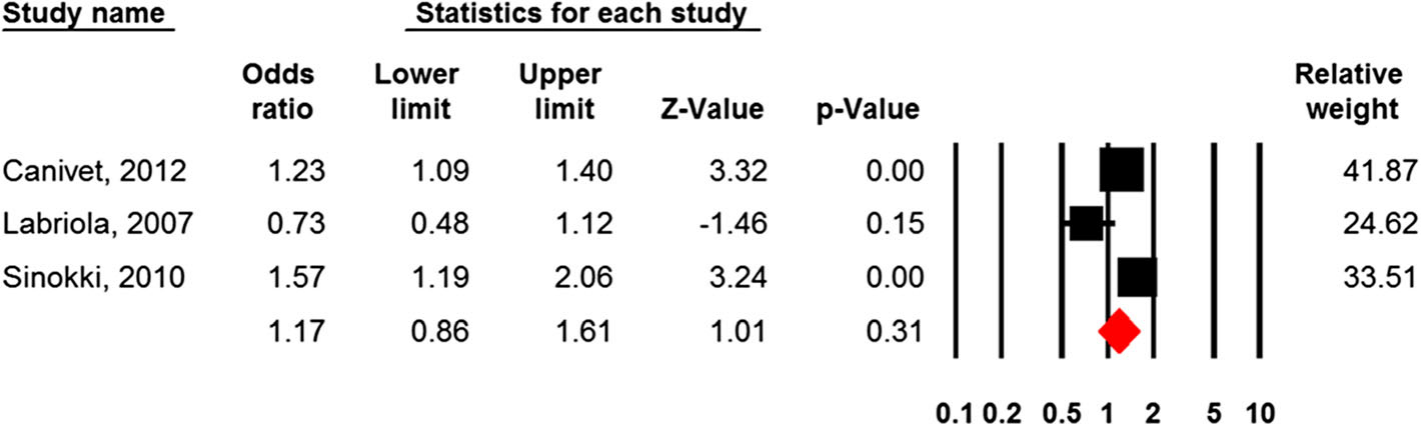
Forest plot Social support

Of the nine studies three were suitable for meta-analysis [53, 54, 71]. Figure 5 and Table 2 shows that aspects of low social support were nonsignificantly associated with subsequent disability retirement (RR = 1.17; 95% CI = 0.86-1.61).

#### Conflicts

Three studies examined the effect of conflicts on subsequent disability retirement (Table 1). Appelberg et al [12] assessed interpersonal conflicts with a single item, whereas Lund et al [63] and Labriola et al [71] assessed conflicts at work with measures derived from the JCQ. All three studies were based on the general working populations of Finland [12] and Denmark [63, 71], and all used registry data to assess disability retirement. Appelberg et al [12] found an increased risk for women, whereas Labriola et al [71] and Lund et al [63] did not find any significant associations between conflicts at work and disability retirement.

#### Harassment

One study of harassment based on the general working population found that harassment did not significantly predict disability retirement when controlling for distress, gender, age, and work exposure factors [80].

### Team climate

Two studies examined the effect of team climate on disability retirement (Table 1). Hinkka et al [59] studied civil-service employees, and assessed team climate based on five items, whereas Ahola et al [48] used data from the general working population, and items from the Healthy Organization Questionnaire. Both studies assessed disability retirement by registries. Hinkka et al found a protective effect of good team climate, whereas no effect was found in the study by Ahola et al.

### Studies of organizational factors

#### Working hours, shift work

Twelve studies addressed work-time schedules [32, 48, 52, 55–57, 59, 60, 62, 64, 65, 72]. Six of these studies compared permanent day workers with shift workers not specifying time of day [56, 57, 59, 60, 62, 64], whereas five studies compared day workers with shift workers specifying evening- or night-shifts [32, 52, 55, 65, 72]. In total, three studies examined the effect of hours worked per week [48, 60, 62].

Three [59, 62, 64] of six [56, 57, 59, 60, 62, 64] studies found significant associations between *shift work* and subsequent disability retirement. One of these studies reported a protective effect of regular shift work compared to day work in the general male working population [62]. Three [52, 55, 65] out of five studies found significant associations for *evening and*/*or night work*.

Of the three studies that examined the effect of hours worked per week [48, 60, 62], the study by Krause et al [62] [48, 60, 62] compared those who worked 60 h or more per week, 45 to 49 h per week, and 40-44 h per week with those who worked less than 40 h per week, respectively. There was a significant increased risk of working 60 h or more per week [62]. Ahola et al [48] and Lahelma et al [60] found no effects of working more than 40 h per week compared to working less.

**Fig. 6:**
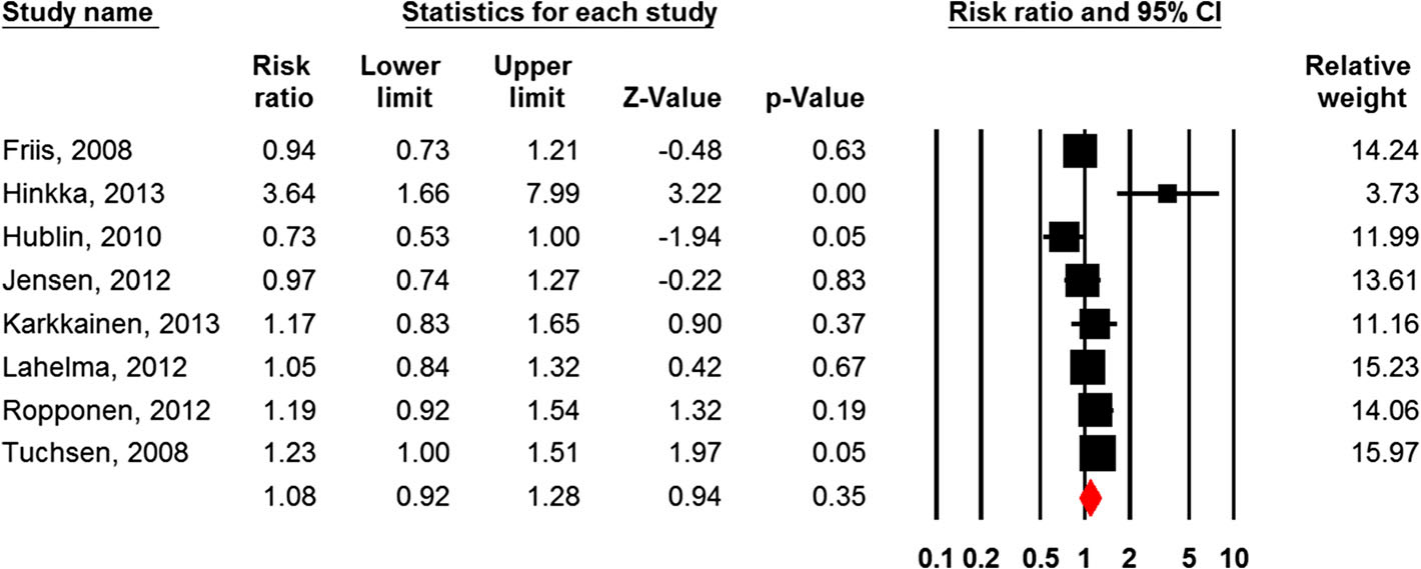
Forest plot Shift Work

Eleven studies compared day workers with evening-, night-, and/or shift workers, eight of which were eligible for meta-analysis [32, 52, 55, 59, 60, 64, 65, 72]. Figure 6 shows nonsignificant effects of shift-work on the risk of subsequent disability retirement (HR = 1.08; 95% CI = 0.92-1.28).

#### Contract type

One study examined the effect of temporary work contracts versus permanent contracts, and reported nonsignificant results [60].

#### Organizational change

Of the 32 studies, just one study addressed organizational change [69]. Based on office staff aged 35-55 working in 20 civil service departments in London, Virtanen et al found that the employees who were transferred to executive agencies compared to those who remained in the Civil Service exhibited increased risk of disability retirement.

#### Downsizing

Only one study addressed downsizing. Vahtera [43] et al found that a reduction in personnel of 18% or more was significantly related to subsequent disability retirement in both male and female municipal employees.

## Discussion

### Summary of evidence

The present systematic critical review found 19 articles of high [12, 32, 43, 47–55, 76–82] and 20 articles of acceptable [56–75] methodological quality. There was moderate evidence for the role of *low control of work situation*: low level of *control* was rather consistently associated with subsequent disability [49–52, 54, 56, 57, 59, 60, 67, 68, 70, 73–75], although five of the 20 studies reported nonsignificant results [55, 63, 66, 71, 74]. Moreover, there was moderate evidence for an effect of the *combination of high quantitative demands and low control* (job strain) (Karasek [3]) predicted subsequent disability [47, 48, 54, 67], although there were two studies (based on one population) with nonsignificant results [49, 79]. These two studies reported significant effectgs of the comniation of low demands and low control, hence control seems to be a decisive factor. There was no major systematic differences in findings between the highest rated and the lowest rated studies that met the criterion for adequate quality.

High *job demands* assessed with validated instruments was reported to predict disability in two studies [54], but nine studies reported nonsignificant findings [49, 55, 60, 63, 66–68, 71]. *Low demands* was found to predict disability awards in two studies [75, 78]. Of the eight studies that measured demands with single items [50, 52, 58, 62, 73–75], two studies [50, 74] reported that “*concentration and attention*” predicted disability. The meta-analysis did not show evidence of effects of high demands on subsequent disability retirement. Hence, with the large number of studies it seems merited to conclude that there is very limited evidence that general job demands predicts disability retirement, while there is limited evidence that the specific factor “*concentration and attention*” does contribute to disability.

There was limited evidence for the association between repetitive work tasks (monotonous work) and disability retirement (one study; Sterud [80]). There was limited evidence for a role of effort reward imbalance and low rewards as predictors of disability pension due to depression (one study; Juvani et al [78]).

Two studies reported that low support from the superior predicted disability pension awards [53, 54], while one [49] study reported that *high* social support predicted disability with mental diagnoses. Five other studies reported no effect of support [59, 60, 63, 68, 71]. Theoretically, one may argue that supportive superiors may facilitate retirement if in the best interest of the employee, or contribute to adjustments of work tasks (demands) to compensate for lower work ability. Hence, social support may affect retirement decisions through several mediation processes.

Interpersonal conflict was associated with disability pension awards in women [12], while two other studies did not find any association between conflicts and disability [63, 71].

Of organizational factors, *downsizing* [43] and *organizational change* [69] predicted subsequent disability retirement. The specific factors “employee development” and “supplementary training” have been reported by one study. These factors may be of significance, but the findings need to be confirmed by other studies and are therefore graded as limited evidence.

There is very limited evidence for *evening and night work*, *shift or period work*, *working hours* >*60 per week*. While three studies found effects of *evening and night work* [52, 55, 65], two did not [32, 72]. Two studies reported effects of *shift or period* –*based work* [59, 64], but four studies did not [56, 57, 60, 62]. The meta-analysis of the effects of shift work on subsequent disability retirement showed nonsignificant results.

While one study found effects of *working hours* >*60 per week* [62], two did not find effects of *working hours* >*40 per week* [48, 60].

Job dissatisfaction and low levels of meaning at work have been reported to predict higher risk of disability retirement ([11]; Clausen et al. [28]) indicating that some of the effects reported here may be mediated through emotional and cognitive factors like attitudes to work and the workplace.

On a theoretical level, we found that task-level, individual-level factors associated with control of one’s work situation was the most consistent significant work factor in processes leading to disability retirement. The level of control depends on the nature of work tasks and how work is organized. Disability is defined as the general inability to perform ones job and one would expect that the demands posed by the work tasks would be paramount in determining retirement due to disability. Surprisingly, psychological job demands was not a consistent predictor of disability awards. Theoretically, high levels of psychological demands may be associated with jobs held by employees with high levels of education and high job involvement [158], hence the appraisal of demands may vary between occupations resulting in inconsistent effect of demands.

### Limitations: Methodological considerations pertaining to primary articles

#### Validity and specificity of exposure variables

The study of psychological and social factors is generally limited to methods based on self-report. Direct observations of working conditions by trained observers are usually not possible as they are very time-consuming. Moreover, the presence of an observer may influence the behavior of those observed.

Many articles presented minimal descriptions of the methods employed to measure psychological and social factors, referring to an instrument, but not specifying which items were used, response scales, or the properties of these.

Of psychological and social factors at work, most studies measured demands, control, and “job-strain”. The *demands* dimension includes several types of demands: Quantitative demands (amount of work, working hours, time pressure) differ from qualitative demands (complexity, standards for quality, problem solving) and emotional demands of dealing with clients, etc. Single-item assessment of demands can only tap into one (narrow) aspect of the many types of demands posed to the employee. Hence, it seems reasonable to recommend restricting conclusions to the specific factor measured rather than making statements pertaining to general job demands. Moreover, findings pertaining to specific factors may direct interventions. The finding that repetitive work tasks may predict disability retirement (Sterud, 2013) indicates that rather concrete organizational interventions may be beneficial.

Similarly, *Control* is a broad dimension that may be defined as the possibility or freedom to choose between alternatives. Control may pertain to control of decisions, breaks, work procedures, working hours (e.g., flexi-time), social interactions with clients or colleagues, etc. Hence, the control dimension incorporates several factors.

Most studies of demands, control, and job strain, were based on the Job Content Questionnaire instrument (JCQ; Karasek et al. [5]), which measures demands by questions pertaining to time pressure, amount of work, and role conflicts. Role conflicts may produce effects on health that differ from those of demands (e.g., Christensen & Knardahl [14, 159]). The control dimension (“*decision latitude*”) of the JCQ includes both “*skill discretion*” (variety of work and opportunity to use skills) and “*decision authority*” (control over decisions that influence work) which may affect health differentially [160]. High levels of skill discretion may imply more interesting work tasks and more responsibility (which may be related to demands). Furthermore, one [42] of two studies that reported skill discretion separately [42, 45] found a significant effect on disability [71]. Neither of these nor a third study from the same research group [37] found significant effects of low decision authority. Control of work time seems to be an important aspect of control [51].

The exposure assessment of studies on shift work was mostly based on dichotomous classification of data ("shift work" compared to "day work"). There is considerable variation in shift-schedule characteristics (e.g., the number of night shifts a year, the speed of shift rotation, regular vs. irregular shift systems, etc.) which may result in misclassification.

Therefore, although some of the most robust findings of the present systematic review pertain to broad dimensions each consisting of several factors; it is possible that applying instruments that measure specific factors would uncover stronger associations with subsequent disability. Some aspects of control may be more important with regard to disability pensioning than others, but this has not been sufficiently examined yet. Moreover, there is a need for studies of more specific exposure factors to allow practical application in interventions or prevention of disability retirement.

Exposure factors may be correlated. Shift-work schedules are most common in occupations in the manufacturing industry and in health-care and nursing where employees are sometimes also exposed to mechanical exposures (like lifting and pushing/pulling objects/patients), chemical exposures, and noise. Hence, many shift-work jobs present a combination of exposures making it difficult to assess the contribution of the shift work schedule *per se*. On the other hand, some organizations with continuous operations carry out most work-intensive procedures during daytime. Hence, some night shift jobs may present low job demands and working with a small group of coworkers. Therefore, drawing general conclusions of effects of shift work based on data from shift schedule with no information of type of work tasks or psychological and social factors is problematic. Conclusions probably only apply to the specific working population investigated and should not be generalized. This was a reason for not including external validity in the assessment of bias in this review. There is a need for studies of effects of interactions of shift characteristics and exposures during work.

#### Validity and specificity of the outcome variable retirement due to disability

The decision to award disability benefit may be based on both assessments of function and on availability of a suitable job. Criteria may vary in emphasis on medical diagnosis or tests of function. Even the support status of the claimant’s partner may be taken into account. To complicate things further, criteria for disability benefits may change over time as a consequence of political decisions. Some countries have introduced temporary disability pension in order to stimulate efforts for rehabilitation/return to work. Moreover, some insurance funds use the label disability compensation for payments of lost wages due to being unable to work due to injury or disease (e.g.,) [161], i.e., synonymously with sickness-absence compensation.

Countries vary in systems and criteria for assigning benefits and compensation to individuals who are no longer able to work. The Nordic countries all provide disability pension based on prolonged sickness absence and failure of retraining (http://www.nordsoc.org/en/) [162], while the Employment and Support Allowance (ESA) of the United Kingdom (UK) and the Social Security Disability Insurance of the United States (US) are based on medical assessments and general function tests [163, 164].

Of the present 39 studies with adequate quality rating, only two investigated non-Nordic populations. This may be a consequence of the differences in public pension systems. The normative age for receiving full pension is higher in the Nordic countries resulting in higher prevalence of disability retirees. Moreover, the public pension systems provide public registries available for scientific study. The age to receive full age pension in Norway is 67 years, 65 in Denmark and Sweden, and 68 in Finland. The state pension age in the UK and Austria is 60 years for women and 65 for men. The standard retirement age is 60 in Italy and until recently, 62 in France. Hence, several factors motivate research on factors determining disability retirement in the Nordic countries. A potential problem with this geographical bias of studies may be that the external validity of conclusions may be limited.

#### Influence of work ability on the perception and reporting of work exposures

Since work ability is a function of the ability of the individual and demands posed by the job, one should expect that individuals with lower work ability may perceive work tasks more demanding. Hence, reporting high levels of job demands may be a consequence of lower work ability, in which case one should expect high job demands to predict disability as a precursor (mediator) rather than as a pathogenic exposure factor.

Therefore, it seems surprising that job demands was not a consistent predictor of disability retirement across studies. Possibly, moderate or high job demands may be associated with interesting and motivating job tasks (and/or higher socioeconomic status), hence high job involvement may buffer effects of high demands in some jobs. Indeed, Blekesaune and Solem [75] reported that “People working in stressful jobs delay nondisability retirement compared with those in less stressful occupations.” An alternative explanation may be that employees with poor health may already have reduced their workload at the time of measuring their work exposures. It should be noted that Samuelsson et al. [48] found that higher levels of job demands was a risk factor of disability pension due to mental diagnoses, hence there may be specific associations between high demands and psychological health problems.

The length of a study follow-up period may influence results. Theoretically, if the follow-up period is short, individuals receiving disability retirement may primarily be employees with somewhat reduced work ability at baseline when the exposure measurements were performed. The present review did not find a systematic tendency of significant results among the studies with the shortest follow-up period. For the control dimension, studies that reported significant effects (control *p* < 0.05; *n* = 18 studies) had a mean follow-up time of 7.6 years (median = 7 years), while studies reporting nonsignificant results (*p* > 0.05; *n* = 6 studies) had a mean follow-up time of 7.8 years (median = 7 years). Studies reporting significant effects of the demand dimension (*p* < 0.05; *n* = 4 studies) had a mean follow-up time of 7 years (median = 6.5 years), while studies reporting nonsignificant results (*p* > 0.05; *n* = 16 studies) had a mean follow-up time of 7.9 years (median = 7.5 years).

### Limitations and strengths of the current review

A major strength of this review was the comprehensive systematic search. Pilot searches showed that it was not possible to cover all psychological, social, organizational exposures by search terms. Therefore, articles were searched based on outcome search terms and all articles investigating/containing organizational, psychological, and social exposures (including moderating factors) were reviewed in full-text. We are therefore reasonably confident that this systematic literature review includes all relevant studies within the time span.

Several studies were identified that measured psychological and social factors, but only entered these data as confounders or moderators in their final analyses. These studies did not allow any conclusions of the contribution of psychological and social factors and consequently failed to reach an acceptable-quality score in this review.

#### Assessment of methodological quality

A second strength of the present review was the specific evaluation of subjective report methods for assessing bias. Most measurements of psychological and social work *exposures* are based on self-reported data. Many studies only report internal consistency (Cronbach’s alpha) and some measure exposures with scaled-down versions of commonly used instruments or with single items with unknown psychometric properties.

There is no universal agreement of criteria for “flawed measurement” of psychological and social factors at work. While many epidemiological studies have measured work exposures with single questions or scales with unknown psychometric properties, experts in psychometrics generally call for the use of multi-question scales that are extensively tested for several aspects of validity, reliability, and item bias in order to conclude that a method is adequate [165]. The present systematic review evaluated bias by a detailed checklist of sources of bias that might originate from subjective-report methods: psychometric quality of instruments (explicit documentation of validity and reliability), analysis of data at organizational unit-level, assessment of traits associated with reporting bias, design with repeated measurements of exposures, and reporting historical exposures. These are methodological issues that influence validity of data, but have not traditionally been considered necessary for publishing epidemiological studies. For the present review, we decided that meeting basic criteria of selection bias and adequate exposure and outcome measurement constituted adequate quality. We found that this corresponded to a quality score of 50%. However, a consequence of including the above-mentioned factors which raises the standards for “perfect methods”, was the broader range of scores (highest score was 81%).

All reviewers noted that some issues were difficult to assess due to inadequate reporting of procedures methods in the primary articles. The checklist was found to be effective in making sure all essential issues were checked in all included primary articles.

There were no major systematic differences in findings between the highest rated and the lowest rated studies that passed the criterion for adequate quality (50%), e.g., the mean internal validity score of the six studies that reported no effects of control was 61%, whereas the mean score of the 18 studies that reported significant effects was 63%. Regarding demands, the mean internal validity score of the 17 studies that reported no increased risk was 62%, whereas the mean score was 63% for the three studies that reported an increased risk.

A third strength of the present review was that the decision to accept methodological quality of a primary study was based on evaluation of its internal validity. External validity determines whom conclusions pertain to, and should not direct conclusions of methodological quality.

The decision of level of evidence was based on GRADE guidelines supplemented with meta analyses. The most completely adjusted effect estimates reported in each individual primary study was entered into the meta analyses, since conclusions of primary articles generally are based on adjusted estimates. It is possible that some studies adjust for factors that are mediators rather than confounders or moderators, e.g., health status. Correcting for mediators may lead to underestimation of the effects of the work related factors. However, it is often not possible to determine if a variable is a mediator with only one or two measurement points. In our opinion, it was not reasonable to compute the combined effect estimates based on the crude effects, given that the outcome is highly related to age.

#### External validity

The aim of the present review was elucidating predictors of permanent disability retirement, thus we did not include studies of employees already on sickness absence or temporary disability. Several countries do not have public systems of disability compensation and several countries do not have registries allowing scientific study of disability benefits. Therefore, one may question the general external validity of the current primary studies. One has to read each study to determine to whom the conclusions apply.

The findings of a study of one type or work or one occupation may not be generalized to other occupations, but may still be of great value. Even findings from a random representative sample in one country may not be generalized to another country with different laws, culture, or compensation systems. The large increase in unemployment in many European countries since 2008 may contribute to differences between countries.

#### Publication bias

The selective publication of studies based on the magnitude and direction of their findings constitute a threat to the validity of meta-analysis [166]. The most consistent finding in the present study was the association between low control and excess risk of subsequent disability retirement. The fail-safe N statistic showed that 623 studies reporting null effects would be needed to attenuate the findings in the present study to non-significant. Hence, the combined effects of control were robust.

### Comparison of findings with previous systematic reviews

We have only found one systematic critical review of psychosocial factors at work as predictors of disability benefits, authored by Dragano and Schneider [167]. With search terms “early retirement”, “premature retirement”, “work disability”, “early pensioning”, “disability pension”, and “disability retirement” their search seems to be somewhat wider than the present review since “work disability” may denote temporary disability. They excluded studies of shift work, their only specified quality criterion was prospective study design, and they did not perform meta-analyses. They concluded that 20 studies met their criteria for inclusion and quality. Six studies in their review did not meet the quality criteria of the present review [95, 98, 99, 101, 104, 111]. Two of these studies reported significant effects of control, demands [99] and job strain [104]. One study [98] reported no effects of control, demands, or opportunities for development. The present review included 25 studies [32, 47–49, 52, 54, 55, 57, 59–61, 64–66, 69, 72–74, 76–82] that were not identified by Dragano and Schneider or that were published after July 31^st^, 2010, the time frame of their search. They reported “Important single factors were low control, monotonous work”, “job strain, effort-reward imbalance”, “lack of social support”, “problems related to the organization of work and to leadership behaviors”.

## Conclusions

The present systematic review showed that psychological and organizational factors at work contribute to early retirement due to disability benefits. There was moderate evidence (i.e., several observational studies of high quality with coherent results) for the role of *low control of work situation* and for the *combination of high quantitative demands and low control* (job strain). There was limited evidence for *downsizing*, *organizational change*, *work demanding attention and concentration, lack of employee development and supplementary training*, *repetitive work tasks*, *low rewards*, *and effort*-*reward imbalance* as predictors of disability, but these findings need replication. There was very limited evidence that general job demands, evening or night work, and low social support from ones superior as predictors of disability retirement.

It seems justified to recommend that managers and leaders intensify their efforts to increase employees’ control of their work situation (decision authority, autonomy), in particular for employees with high levels of job strain. During periods of downsizing and organizational change, managers should pay attention to processes that may facilitate disability. Employee development and supplementary training may be particularly important measures to maintain competence/work ability in a working life with rapidly changing technology and demands.

### Research needs

Most of the reviewed studies applied measurement instruments that measure broad dimensions combining factors that may have different effects on health-related outcomes. Most studies of job demands, control, and the combination of demands and control (job strain), have applied the JCQ [5]. This instrument includes role conflict in job demands and both decision authority and skill discretion under the control dimension. Therefore, studies conducted by the JCQ may underestimate effects if only one of these factors contribute to disability retirement. There is a need for studies based on measurements of specific work exposures. Furthermore, knowledge of risks (or protective factors) must be specific in order to direct design of interventions or prevention. It seems merited to recommend measurement of specific exposure factors in future studies of disability.

Almost all studies found were conducted with Nordic populations. In order to take cultural, political, and economic aspects into account, there is a need for studies of employees from non-Nordic countries. The challenges posed by the combination of current high rates of unemployment in young Europeans and the demographic shift to aging populations, raises the need for knowledge of trajectories of competence/work ability and exit from working life, and the influence of work factors on these trajectories.

## Additional file

Additional file 1:
**Table A**: Search strategy; **Table B**: Quality assessment check list; **Table C**: Excluded studies - characteristics and findings; **Table D**: Scores of internal validity for accepted studies; **Table E**: Scores of internal validity for studies that were excluded by quality criteria. (DOCX 97 kb)

## Abbreviations

COPSOQ: The Copenhagen Psychosocial Questionnaire
ERI: Effort-reward imbalance model
GRADE: The Grading of Recommendations Assessment, Development, and Evaluation Working Group
HR: Hazard ratio
JCQ: The Job content questionnaire
OR: Odds ratio
RR: Risk ratio
