# Supplementary material for: The contribution from psychological, social, and organizational work factors to risk of disability retirement: a systematic review with meta-analyses
Source: BMC Public Health. 2017 Feb 8;17:176. doi: 10.1186/s12889-017-4059-4 (PMC5299735; doi:10.1186/s12889-017-4059-4)
Supplement: Additional file 1: — Table A: Search strategy; Table B: Quality assessment check list; Table C: Excluded studies - characteristics and findings; Table D: Scores of internal validity for accepted studies; Table E: Scores of internal validity for studies that were excluded by quality criteria. (DOCX 97 kb) [file 12889_2017_4059_MOESM1_ESM.docx]

**Additional file 1**

**Supporting Information contents:**

Table A: Search strategy

Table B: Quality assessment check list

Table C: Excluded studies: characteristics and findings

Table D: Scores of internal validity for accepted studies

Table E: Scores of internal validity for the studies which were excluded by quality criteria

**Table A: Search strategy**

| Database | Medline | Embase | PsychINFO |
| --- | --- | --- | --- |
| Search strings | **1.** ((Disability or invalid* or incapacity) adj3 (pension* or retirement or insurance or allowance? or compensation? or award* or benefit? or work*)).tw.  **2.** pensions/  **3**. retirement/  **4**. (Disability or invalid* or incapacity).mp.  **5**. 2 or 3  **6.** 4 and 5  **7.** exp insurance, disability/  **8.** disabled persons/  **9.** employment/  **10.** or/7-9  **11.** (pension* or retirement).mp.  **12.** 10 and 11  **13.** 1 or 6 or 12 | **1**. ((Disability or invalid* or incapacity) adj3 (pension* or insurance? or retirement or allowance? or compensation? or award* or benefit? or work*)).tw.  **2**. pension/  **3.** exp health insurance/  **4.** retirement/  **5**. workman compensation/  **6.** medical leave/  **7.** or/2-6  **8.** (Disability or invalid* or incapacity).mp.  **9.** 7 and 8  **10.** disabled person/  **11.** exp disability/  **12.** 10 or 11  **13.** (pension* or retirement).mp. [mp=title, abstract, subject headings, heading word, drug trade name, original title, device manufacturer, drug manufacturer, device trade name, keyword]  **14**. 12 and 13  **15.** 1 or 9 or 14  **16.** limit 15 to embase | **1.** ((Disability or invalid* or incapacity) adj3 (pension* or insurance? or retirement or allowance? or compensation? or award* or benefit? or work*)).tw.  **2.** exp Employee Benefits/  **3.** exp Employment Status/  **4.** personnel termination/  **5.** Retirement/  **6.** or/2-5  **7.** (Disability or invalid* or incapacity).mp.  **8.** 6 and 7  **9.** exp Disabilities/  **10**. Disabled Personnel/  **11**. 9 or 10  **12**. (pension* or retirement).mp.  **13.** 11 and 12  **14.** 1 or 8 or 13 |
|  |  |  |  |
|  |  |  |  |
|  |  |  |  |
|  |  |  |  |
|  |  |  |  |
|  |  |  |  |
|  |  |  |  |
|  |  |  |  |
|  |  |  |  |
|  |  |  |  |
|  |  |  |  |
|  |  |  |  |

Note: Abbrevations

Adj3= the words have to appear within 3 words of each other

/ = MeSH term

* = truncation (unlimited amount of signs)

? = truncation (0 or 1 sign)

.tw = text words, search looks in title and abstract

.mp = multiple purpose, search looks in Title, Original Title, Abstract, Subject Heading, Name of Substance, and Registry Word fields.

**Table B: Quality assessment check list**

| **Quality assessment of primary studies: Prospective design** | | |  |  |  |  |  |  |  |  |
| --- | --- | --- | --- | --- | --- | --- | --- | --- | --- | --- |
| Refman ID-nr: ________ Author: ___________________________ Journal _____________________________________ | | | | | | | |  |  |  |
| Title: ___________________________________________________ Evaluated by: _________________ date: _________ | | | | | | | |  |  |  |
| Schematic assessment of methodological quality of included articles (**na** = not applicable). | | |  |  |  |  |  |  |  |  |
| **#** |  | **Quality assessment item list** | **SCORE** | | | | **SUM** | **TOTAL** | **%** | Max possible score |
|  | **Pros** | **Internal validity** |  |  |  |  |  |  |  |  |
| **S** | **Pros** | **Study population - subjects:** | **3** | **2** | **1** | **0** |  |  |  |  |
| 1 |  | Positive if the main feature (type of work, description of sampling frame, and distribution by age and gender) of the study population were stated: ***1*** |  |  |  |  |  |  |  |  |
| 2 |  | Selection bias: Positive if possible problems with selection bias in recruitment are addressed and avoided? *Non-responder analysis with no evidence of selection bias:* ***2****; No obvious major recruitment selection problems:* ***1****; No information:* ***0*** |  |  |  |  |  |  |  |  |
| 3 |  | Selection bias: Positive if exclusion criteria are specific and do not allow selection bias: ***1*** |  |  |  |  |  |  |  |  |
| 4 |  | Positive if the response rate at follow-up was adequate: *>85% of included subjects (responded at baseline):* ***3****; 75–84%:* ***2****; 50–74%:* ***1****, <50%:* ***0*** |  |  |  |  |  |  |  |  |
| 5 |  | Positive if attrition analysis was performed: *No or little bias: 2, predictors of attrition adequately discussed:* ***1*** |  |  |  |  |  |  |  |  |
| 6 | na | Selection bias: *Control group:* ***1*** |  |  |  |  |  |  |  |  |
| 7 | na | Selection bias: Positive if randomization to treatment/intervention/exposure: ***3*** |  |  |  |  |  |  |  |  |
| 8 |  | Positive if treatment/intervention/exposure is compared with other treatments/interventions/exposures (not only no-treatment control group): ***1*** |  |  |  |  |  |  |  |  |
| 9 |  | Cross-over design: ***1*** |  |  |  |  |  |  |  |  |
| 10 | na | Positive if the cases and referents were drawn from the same population: ***2*** |  |  |  |  |  |  |  |  |
| 11 | na | Positive if a clear definition of the cases and referents was stated: ***1*** |  |  |  |  | **0** |  |  | 9 |
| **E** | **Pros** | **Exposure measurements:** | **3** | **2** | **1** | **0** |  |  |  |  |
| 1 |  | Positive if the assessed psychological/social/organizational exposure factors were explicitly defined or sample items presented: ***1*** |  |  |  |  |  |  |  |  |
| 2 |  | Positive if psychosocial/organizational exposures were assessed several times: *>3 times:* ***3****; 3 times:* ***2****; 2 times:* ***1*** |  |  |  |  |  |  |  |  |
| 3 |  | Positive if psychosocial/organizational exposures were assessed by instruments that have been tested for validity and reliability with reference to psychometric data or to article with tests: *All factors have good psychometric quality:* ***3****; Some factors have good psychometric quality:* ***2;*** *Some factors have been tested:* ***1.*** (reporting only Cronbach's alphas is not adequate) |  |  |  |  |  |  |  |  |
| 4 |  | Positive if higher level of measurement scale for psychosocial/organizational exposure were used in the analyses: *Interval or ratio scale or rank scale (e.g. high, medium, low):* ***2****; Dichotomous classification:* ***1*** |  |  |  |  |  |  |  |  |
| 5 |  | Positive if psychosocial/organizational exposures (of each subject) were assessed by external observation, records, or registry data of verified quality and used in the analysis: ***2*** |  |  |  |  |  |  |  |  |
| 6 |  | Positive if exposure data were aggreagated to unit level (department, company): ***1*** |  |  |  |  |  |  |  |  |
| 7 | na | Positive if exposures were measured in identical way among the cases and referents: ***1*** |  |  |  |  |  |  |  |  |
| 8 |  | Positive if data on historical exposures at work were included in the analysis: ***1*** |  |  |  |  | **0** |  |  | 13 |
| **O** | **Pros** | **Outcome measurement:** | **3** | **2** | **1** | **0** |  |  |  |  |
| 1 |  | Positive if there is a specific definition of criteria for outcome (case definition): ***2*** |  |  |  |  |  |  |  |  |
| 2 |  | Positive if data on outcome were collected using explicitly described methods of acceptable quality: ***1*** |  |  |  |  |  |  |  |  |
| 3 |  | Positive if outcome was based on registry data of verified quality: ***2*** |  |  |  |  |  |  |  |  |
| 4 | na | Positive if outcome is based on examination by third party (e.g. doctor) that is blinded to exposure status: ***1*** |  |  |  |  |  |  |  |  |
| 5 |  | Positive if outcome was measured several times for each subject: *2 or more outcome measurement times:* ***1****; 1 sample time point:* ***0*** |  |  |  |  |  |  |  |  |
| 6 | na | Positive if incident cases were used (prospective enrolment): ***1*** |  |  |  |  | **0** |  |  | 6 |
| **A** | **Pros** | **Analysis and data presentation:** | **3** | **2** | **1** | **0** |  |  |  |  |
| 1 |  | Positive if the statistical models used were appropriate for the outcome studied and the measurement of the association estimated with the models were presented (including confidence intervals): ***2*** |  |  |  |  |  |  |  |  |
| 2 |  | Positive if analyses explicitly test confounding or moderation: ***1*** |  |  |  |  |  |  |  |  |
| 3 |  | Positive if crude models were presented: ***1*** |  |  |  |  |  |  |  |  |
| 4 |  | Positive if the number of cases in the multivariate analysis was at least 10 times the number of independent variables in the analysis: ***1*** |  |  |  |  | **0** |  |  | 5 |
| **C** | **Pros** | **Confounders (factors that affect both exposures and outcomes)** | **3** | **2** | **1** | **0** |  |  |  |  |
| 1 |  | Positive if analyses are controlled for (stratified or adjusted) data on health status and work ability at baseline: ***2*** |  |  |  |  |  |  |  |  |
| 2 |  | Positive if the study controlled for (stratified or adjusted) confounding factor: ***Age****:* ***2*** |  |  |  |  |  |  |  |  |
| 3 |  | Positive if the study controlled for confounding factor: ***Gender: 2*** |  |  |  |  |  |  |  |  |
| 4 |  | Positive if the study controlled for (stratified or adjusted) confounding factor: ***Education level: 1*** |  |  |  |  |  |  |  |  |
| 5 |  | Positive if the study controlled for (stratified or adjusted) confounding factor: ***Income: 1*** |  |  |  |  |  |  |  |  |
| 6 |  | Positive if the study controlled for (stratified or adjusted) confounding factor: ***Occupational class: 1*** |  |  |  |  |  |  |  |  |
| 7 |  | Positive if subjects are uninformed about hypotheses tested by the study: ***2*** |  |  |  |  |  |  |  |  |
| 8 |  | If both exposures and outcome measured by questionnaires: Positive if the study controlled for confounding factor: ***Traits that may influence reporting bias (e.g. neuroticism): 2*** |  |  |  |  | **0** |  |  | 13 |
|  |  | **TOTAL scores** |  |  |  |  |  | 0 | **0** | 46 |
|  |  |  |  |  | | |  |  |  |  |
| **EV** | **Pros** | **External validity** | **3** | **2** | **1** | **0** |  |  |  |  |
| 1 |  | Positive if study population is representative of a defined working population (for a defined type of work, a defined branch, or for any type of work, etc): *Specific inclusion criteria for a defined working population:* ***1*** |  |  |  |  |  |  |  |  |
| 2 |  | Positive if study population is representative for a defined working population (for a defined type of work, a defined branch, or for any type of work, etc): *Subjects recruited from >2 organizations/units:* ***2****; Subjects recruited from 2 organizations / units:* ***1*** |  |  |  |  |  |  |  |  |
| 3 | na | Sample size: 500: ***2***; 50–499: ***1***; <50: ***0*** |  |  |  |  |  |  |  |  |
| 4 | na | Sample size, number of cases: *50: 2; 25–49:* ***1****; <25:* ***0*** |  |  |  |  |  |  |  |  |
| 5 |  | Positive if the participation rate at the beginning of the study was adequate: *85%:* ***3****; 75-84%:* ***2****; 50-74%:* ***1****; < 50%:* ***0*** |  |  |  |  | **0** |  |  |  |
|  |  | **TOTAL scores** |  |  | | |  | 0 | **0** | 6 |
|  |  |  |  |  | | |  |  |  |  |
| **M** | **Pros** | **Moderators (factors that may affect associations between exposures and outcome)** |  |  |  |  |  |  |  |  |
| MW | **Pros** | **Other types of exposures at work** | **3** | **2** | **1** | **0** |  |  |  |  |
| 1 |  | Positive if representative level of physical exposure *intensities* at work was measured and used in the analyses? *By measurements, objective observation or records:* ***2****; By questionnaires:* ***1****; Not assessed:* ***0*** |  |  |  |  |  |  |  |  |
| 2 |  | Positive if representative level of physical exposure *durations* at work was measured and used in the analyses? *By measurements, objective observation or records:* ***2****; By questionnaires:* ***1****; Not assessed:* ***0*** |  |  |  |  |  |  |  |  |
| 3 |  | Was duration of occupation with physical exposures reported? *Yes:* ***1****; No:* ***0*** |  |  |  |  |  |  |  |  |
| 4 |  | Positive if higher level of measurement scale for physical exposure were used in the analyses: *Interval or ratio scale:* ***3****; Rank scale (f. ex. high, medium, low):* ***2****; Dichotomous scale:* ***1****; Not quantified:* ***0*** |  |  |  |  |  |  |  |  |
| 5 |  | Positive if representative level of chemical or biological exposure *intensities* at work was measured and used in the analyses? *By measurements, objective observation or records:* ***2****; By questionnaires:* ***1****; Not assessed:* ***0*** |  |  |  |  |  |  |  |  |
| 6 |  | Positive if representative level of chemical or biological exposure *durations* at work was measured and used in the analyses? *By measurements, objective observation or records:* ***2****; By questionnaires:* ***1****; Not assessed:* ***0*** |  |  |  |  |  |  |  |  |
| 7 |  | Was duration of occupation with chemical or biological exposures reported? *Yes:* ***1****; No:* ***0*** |  |  |  |  |  |  |  |  |
| 8 |  | Positive if higher level of measurement scale for chemical or biological exposure were used in the analyses: *Interval or ratio scale:* ***3****; Rank scale (f. ex. high, medium, low):* ***2****; Dichotomous scale:* ***1****; Not quantified:* ***0*** |  |  |  |  | 0 |  |  | 16 |
| ML | **Pros** | **Leisure-time exposures** | **3** | **2** | **1** | **0** |  |  |  |  |
| 9 |  | Positive if data on family situation were used in the analysis: ***1*** |  |  |  |  |  |  |  |  |
| 10 |  | Positive if data on subjects' economical situation were used in the analysis: ***1*** |  |  |  |  |  |  |  |  |
| 11 |  | Positive if data on physical activity during leisure time were used in the analysis: ***1*** |  |  |  |  |  |  |  |  |
| 12 |  | Positive if study controlled for ***Insomnia or sleep problems: 1*** |  |  |  |  |  |  |  |  |
| 13 |  | Positive if the study controlled for ***Smoking: 1*** |  |  |  |  |  |  |  |  |
| 14 |  | Positive if the study controlled for ***Overweight: 1*** |  |  |  |  | 0 |  |  | 6 |
|  |  | **TOTAL scores** |  |  | | |  | 0 |  |  |

**Table C: Excluded studies: characteristics and findings**

| **Authors, year publication,**  **Country (subjects studied)** | **Exposures investigated** | **Employee groups (types of work), number of subjects** | **Outcomes (definition of disability)** | **Conclusions**  **(with OR, RR, HR)** | **Control for confounders** |
| --- | --- | --- | --- | --- | --- |
| Alavinia et. al., 2009,  The Netherlands [102] | Job demands; skill discretion; job control | Construction workers, n=850 (40 cases) | Disability pension awards, questionnaire | **Crude estimates only**  High job demands: Crude HR= 1.24 (0.66-2.32)  Low job control: Crude HR=0.85 (0.45-1.64)  Low skill discretion: Crude HR=1.63 (0.85-3.14) | Age, white-collar work, mechanical work factors, work ability |
| Albertsen et. al., 2007,  Denmark [105] | Decision authority, social support, job insecurity, role conflict, interpersonal conflicts, sexual harassment, violence and threats of violence, teasing, psychological demands, responsibility, concentration | General working population, n=5940 (87 cases) | Disability pension awards, registry | **Crude estimates only**  *Women*:  Job insecurity (scale): **HR=1.10** (1.01-1.21)  Decision authority (scale): **HR=0.90** (0.81-1.00)  Role conflicts (scale): HR=1.05 (0.92-1.20)  Social support (scale)**: HR=0.85** (0.76-0.96)  High psychological demands: HR=1.84 (0.96-3.53)  High responsibility: HR=1.42 (0.72-2.81)  High concentration : HR=1.64 (0.89-3.00)  Conflicts: HR=0.75 (0.34-1.65)  Violence: HR=1.84 (0.57-5.88)  Teasing: HR=0.89 (0.12-6.40)  Sexual harassment: HR=2.35 (0.57-9.62)  *Men*  Job insecurity (scale): HR=1.10 (0.97-1.25)  Decision authority (scale): HR=0.90 (0.78-1.05)  Role conflicts (scale): HR=1.04 (0.87-1.-1.23)  Social support (scale): HR=1.04 (0.87-1.26)  High psychological demands: HR=0.70 (0.24-2.02)  High responsibility: HR=1.05 (0.45-2.43)  High concentration : HR=0.98 (0.39-2.49)  Conflicts: HR=1.31 (0.53-3.19)  Violence: HR= no men exposed  Teasing: HR=no men exposed  Sexual harassment: HR=no men exposed | Sex, age, occupation, social class, health behavior, family status |
| Allaire et al, 2009, USA [149] | Work hours per week; Coworker/supervisor support; Stressful job | Nested case control design: 231 cases with Rheumatoid Arthritis and  722 controls | Disability pension awards, questionnaire | **Crude estimates only**  Work hours per week (continuous):  **OR=0.9 (0.8-0.9)**  Coworker/supervisor support(ref= most or all the time): OR=0.9 (0.6–1.4)  Stressful Job (ref=no): OR=1.4 (0.9-2.3) | Age, gender, RA disease, general health, mechanical factors at work |
| Beiring-Sørensen et.al, 1999, Denmark [115] | Problems at work; work in 3 shifts; variation at work; own influence at work; work environment; speed at work, satisfied with work | General working population, n=892 (cases=84) | Disability pension awards, registry | **Estimates were not reported**  No significant organizational and psychosocial factors | General health, occupational conditions, leisure time factors, stimulants, sleep pattern |
| Haaramo et. al., 2011, Finland [44] | Shift work; working overtime; psychosocial job strain | General working population, n=6042 (cases=561) | Disability pension awards, registry | **Estimates were not reported** |  |
| Hasle et at, 1989, Denmark [127] | Rapid work pace | Blue- collar workers:  Control group n=1500;  Case group n=581 | Premature pensioning, registry | **Crude estimates only**  Rapid work pace  Cases= 46.8% vs. controls=36.8% (p<0.01) |  |
| Haukenes et. al., 2011, Norway [90] | Working hours; Job demands; Job control; | General working population, n=7031 (cases=209) | Disability pension awards, registry | **Crude estimates only**  Decreasing levels of job control was associated with disability |  |
| Johansson et al, 2012, Sweden [87] | Job control | Cohort of 49321 men (cases=1977) | Disability pension awards, registry | **Crude estimates only**  Low job control (ref=high):  **HR=3.66 (3.16-4.24)** |  |
| Labriola et. al., 2009 , Denmark [99] | Influence; information | General working population, n=8475, | Disability pension awards, registry | **Estimates were not reported** | Age, sex, BMI, ergonomic work environment |
| Lallukka et. al., 2010, Finland [45] | Job strain; working overtime; shift work | General working population, n=5986 (cases=457) | Disability pension awards, registry | **Estimates were not reported** | Age, sex, marital status, general health, health behavior, occupation, sleep problems |
| Leinonen et. al., 2011, Finland [89] | Job control; Job demands; shift work; working overtime; workplace bullying | General municipal employees, n=6516 (cases=600) | Disability pension awards, registry | **Estimates were not reported** |  |
| Lund et. al., 2010, Denmark [95] | Decision authority; information | General working population, n=8287 (cases=346) | Disability pension awards, registry | **Estimates were not reported** | Age, sex, smoking, BMI, self rated health, physical and ergonomic work factors |
| Månsson et. al., 1998, Sweden [116] | Discomfort at work; shift work | General male working population, n=5782 (cases=715) | Disability pension awards, registry | **Crude estimates only**  Discomfort at work (Yes/no): HR=**1.8** (1.5-2.3)  Shift work (Yes/no): HR=0.9 (0.8-1.1) |  |
| Nilsen et. al., 2012, Norway [146] | Concentration and attention ;demanding work; job satisfaction | General male working population, n=32948 (cases=1848) | Disability pension awards, registry | **Estimates were not reported** | Age, sex, occupational factors, psychosocial factors, health conditions, behavioral factors, educational level, illness |
| Pietilainen et. Al.,2011, Finland [46] | Shift work; Temporary work contract; Working overtime; Job control; Job demands; social support | General municipal employees, n=6525 (cases=625) | Disability pension awards, registry | **Estimates were not reported**  Working conditions explained about 20 percent of the  association of self-rated health with subsequent disability retirement  due to all causes |  |
| Saastamionen et. al., 2011, Finland [147] | Social support; Job strain | General municipal employees, n=6258 (cases=594) | Disability pension awards, registry | **Crude estimates only**  *Social support* (ref=high)  Medium: HR=1.27  Low: HR=1.64  *Job strain* (ref=low)  Active: HR=1.12  Passive: HR=1.45  High: HR=1.91 |  |
| Salo et. al., 2010, Finland [93] | Shift work | Public sector employees, n=56732 (cases=4028) | Disability pension awards, registry | **Estimates were not reported** | age, sex, socioeconomic status, smoking, alcohol intake,  body mass index, physical activity, diagnosed somatic disease, use of pain killers, depression, anxiety, and use of anxiolytics |
| Salonen et al, 2003, Finland [111] | Job stress and demands | 126 ageing food industry employees (cases 49) | Disability pension awards, unemployment pension, unemployment, and death, registry | **Crude estimates only**  Job stress and demands p<0.96 | Short report |
| Sivertsen et. al., 2006, Norway [106] | Shift work | General working population, n=37308 (cases=915) | Disability pension awards, registry | **Crude estimates only**  Shift work (ref=no): HR=0.80 |  |
| Stattin et. al., 2005, Sweden [108] | Job control; Work demand; Social support | Construction workers, n=87000 (cases=6000) | Disability pension awards, registry | **Crude estimates only**  *Job control* (ref=often)  Rather often: OR= 1.13 (1.05-1.21)  Sometimes: OR=1.46 (1.34-1.58)  Rather seldom: OR=1.44 (1.21-1.71)  Seldom: OR=1.86 (1.58-2.18)  *Work demand* (ref=low)  High: OR=2.64 (1.72-4.06)  Low control/high demands: OR=4.1 (2.9-5.8)  Social support: no clear association | Age, sex |
| Tuomi et. al., 1991, Finland [126] | Work load (median high/low) | Municipal employees, n=6165 (cases=544) | Work disability, questionnaire | **Estimates were not reported** |  |
| Tuomi et. al., 1991, Finland [154] | Mental demands; Work schedule; Social organization; Possibilities for development | General municipal employees, n= 6257 (cases=544) | Disability pension awards, registry | **Crude estimates only**  Disability due to mental disease  Men  Mental demands: RR=1.6  Work schedule: RR=1.8  Social organization: RR=1.5  Possibilities for development: RR=1.3  Women  Mental demands: RR=1.4  Work schedule: RR=3.1  Social organization: RR=2  Possibilities for development: RR=1.0 | No statistical test of significance |
| Turner et. al., 2007, USA [104] | Hectic work; Working very fast; Excessive work; Supervisor listen; Take breaks when wants; Satisfaction | Washington State workers, n=2055 (cases=899) | 180 days wage replacement, total temporary disability compensation, registry | **Crude estimates only**  *Hectic work* (ref=disagree)  Agree: OR=**1.79** (1.16-2.78)  *Work very fast* (ref=disagree)  Agree: OR=**2.07** (1.20-3.57)  Strongly agree: OR=**3.11** (1.84-5.26)  *Excessive work* (ref=disagree)  Agree: OR= 1.44 (0.97-2.13)  Strongly agree: OR=**2.85** (1.91-4.23)  *Supervisor listen* (ref=agree)  Disagree: OR=**1.73** (1.21-2.46)  *Breaks* (ref=agree)  Disagree: OR=1.32 (0.96-1.81)  *Satisfaction* (very/somewhat satisfied)  Not very satisfied: OR=**1.77** (1.20-2.62) |  |
| van den Berg et. al., 2010,  Europe [92] | Job control; Effort reward imbalance; Time pressure | General working population, n=4611 | Disability pension awards, questionnaire | **Estimates were not reported** |  |

**Table D: Scores of internal validity for accepted studies**

| **Authors, year publication,**  **Country (subjects studied)** | **Exposures investigated (instruments used)** | **Internal validity score** |
| --- | --- | --- |
| Ahola et. al., 2011, Finland[[20](#_ENREF_20)] | Weekly hours; job strain (JCQ) (no test of control and demands); team climate (HOQ); job insecurity | 73% |
| Appelberg et. al., 1996, Finland[[22](#_ENREF_22)] | Interpersonal conflict at work | 66% |
| Blekesaune et. al., 2005, Norway[[79](#_ENREF_79)] | Job stress (two single items); Decision authority (two single items) (job exposure matrix, no validated instruments) | 50% |
| Brage et. al., 2007, Norway[[65](#_ENREF_65)] | Organizational job stress; psychological job stress | 61% |
| Canivet et. al., 2012, Sweden[[58](#_ENREF_58)] | Job demands (JCQ), decision latitude (JCQ), job strain (JCQ), job support (Swedish version of JCQ) | 68% |
| Christensen et. al., 2008, Denmark[[74](#_ENREF_74)] | Decision authority (single item); information; variation in work | 57% |
| Clausen et. al., 2014, Denmark[[80](#_ENREF_80)] | Influence at work (four items), Quality of leadership (four items), Work pace (single item), Quantitative demands (single item) All items from COPSOQ | 67% |
| Claussen et. al., 2009, Norway[[60](#_ENREF_60)] | Job control (decision authority); shift work | 64% |
| Claussen et. al., 2009, Norway[[61](#_ENREF_61)] | Job control (decision authority); shift work | 64% |
| Falkstedt et. al., 2014, Sweden[[81](#_ENREF_81)] | Job control (job exposure matrix – decision authority/skill discretion) | 68% |
| Friis et. al., 2008, Denmark[[56](#_ENREF_56)] | Working schedule; work speed/pressure; busy at work; influence at work (single item) | 68% |
| Hagen et. al., 2002, Norway[[62](#_ENREF_62)] | Excessive job demands, authority to plan own work, concentration and attention | 64% |
| Hagen et. al., 2006,  Norway[[78](#_ENREF_78)] | Concentration and attention; Stress and tension; Authority to plan own work (single item) | 52% |
| Hinkka et. al., 2013, Finland[[63](#_ENREF_63)] | Shift work, work control (single item; decision authority), opportunities for mental growth, support from supervisor, feedback from supervisor | 64% |
| Holmberg et. al., 2006, Sweden[[70](#_ENREF_70)] | Job demands (JCQ); decision latitude (JCQ) | 59% |
| Hublin et. al., 2010, Finland[[47](#_ENREF_47)] | Shift work | 68% |
| Jensen et. al., 2012, Denmark[[59](#_ENREF_59)] | Decision latitude (JCQ), demands (JCQ, the Danish version), shift work | 68% |
| Juvani et. al., 2014, Finland[[82](#_ENREF_82)] | Effort-reward imbalance (one item on effort and three items on rewards, adapted from the standard 10 item ERI scale developed by J. Siegrist) | 81% |
| Karkkainen et. al., 2013, Finland[[69](#_ENREF_69)] | Work-time schedules (day, night, evening, shift work) | 61% |
| Krause et. al., 1997, Finland[[66](#_ENREF_66)] | Weekly work hours; Overwork index; Shift work; Deadlines (ref=weekly or less, daily or more often) | 61% |
| Krokstad et. al., 2002, Norway[[54](#_ENREF_54)] | Job control (decision authority); high demands in concentration and attention | 70% |
| Labriola et. al., 2007, Denmark[[75](#_ENREF_75)] | Decision authority (ref=high); skill discretion (ref=high); social support (ref=high); conflicts (ref=low); psychological demands (ref=low) (JCQ) | 57% |
| Lahelma et. al., 2012, Finland[[64](#_ENREF_64)] | Work arrangements (shift work; temporary work contract; working overtime (hours>40per week)), decision latitude (JCQ), job demands (JCQ), social support (Sarason) | 64% |
| Laine et. al., 2009, Finland[[71](#_ENREF_71)] | Job strain (JCQ), job control, job demands (JCQ) | 59% |
| Lund et. al., 2001,  Denmark[[72](#_ENREF_72)] | Job demands,  Decision authority; Social support; Skill discretion (JCQ, 20 items) | 59% |
| Lund et. al., 2003, Denmark[[67](#_ENREF_67)] | Decision authority; job demands; social support; conflict at work;  employee development; supplementary training (JCQ: 18 items) | 61% |
| Mantyniemi et. al., 2012, Finland[[52](#_ENREF_52)] | Job strain based on work unit and occupational title, respectively (derived from JCQ; conflicting demands not included)(no test of demands and control) | 75% |
| Robroek et. al., 2013, European countries[[77](#_ENREF_77)] | High time pressure (single item); low decision latitude (JCQ: two items, one on authority and one on skill discretion); low rewards (JCQ) | 57% |
| Ropponen et. al., 2012,  Finland[[76](#_ENREF_76)] | Work-time schedules (day, shift, evening/night) | 57% |
| Ropponen et. al., 2013, Sweden[[83](#_ENREF_83)] | Job demands (5 items); job control (7 items); social support at work (4 items); job strain | 71% |
| Samuelsson et. al., 2012, Sweden[[53](#_ENREF_53)] | Job demands (continuous); job control (continuous); Social support (continuous); job strain (validated JEM, based on principal component factor analysis of the Swedish  questionnaire items relating to work) | 73% |
| Sinokki et.al., 2010,  Finland[[57](#_ENREF_57)] | Social support at work, supervisor and coworkers (JCQ) | 68% |
| Sterud T. al., 2013, Norway[[84](#_ENREF_84)] | Job demands (single item), Job control (three items), supportive leadership (three items), bullying/harassment (three items), monotonous work (single item) Some items have been tested for psychometric quality | 61% |
| Støver et. al., 2013, Norway[[85](#_ENREF_85)] | Cumulative summation index of 11 psychosocial work exposure questions | 75% |
| Thielen et. al., 2013, Denmark[[86](#_ENREF_86)] | Mental demands (single item, COPSOQ) | 77% |
| Tüchsen et. al., 2008,  Denmark[[68](#_ENREF_68)] | Shift workers compared with permanent day workers | 61% |
| Vahtera et. al., 2005,  Finland[[49](#_ENREF_49)] | Downsizing (ref=reductions in personnel less than 8% vs.  minor downsizing (8%– 18%) and major downsizing (more than 18%). | 77% |
| Vahtera, J. et. al., 2010, Finland[[55](#_ENREF_55)] | Self-assessed worktime control (7 items scale); Co-worker assessed worktime control (7 items scale) (Validated instrument) | 70% |
| Virtanen et.al., 2010  UK[[73](#_ENREF_73)] | Organizational change | 59% |

**Table E: Scores of internal validity for studies that were excluded by quality criteria**

| **Authors, year publication,**  **Country (subjects studied)** | **Exposures investigated** | **Quality score internal validity** |
| --- | --- | --- |
| Alavinia et. al., 2009,  The Netherlands | Job demands; skill discretion; job control | 48% |
| Albertsen et. al., 2007,  Denmark | Decision authority, social support, job insecurity, role conflict, interpersonal conflicts, sexual harassment, violence and threats of violence, teasing, psychological demands, responsibility, concentration | 48% |
| Allaire et al, 2009, US | Work hours per week; Coworker/supervisor support; Stressful job | 18% |
| Beiring-Sørensen et.al, 1999, Denmark | Problems at work; work in 3 shifts; variation at work; own influence at work; work environment; speed at work, satisfied with work | 11% |
| Haaramo et. al., 2011, Finland | Shift work; working overtime; psychosocial job strain | 43% |
| Hasle et at, 1989, Denmark | Rapid work pace | 17% |
| Haukenes et. al., 2011, Norway | Working hours; Job demands; Job control; | 48% |
| Johansson et al, 2012, Sweden | Job insecurity (JCQ), Decision authority (JCQ), Role conflicts (JCQ), Social support (JCQ), High psychological demands, High responsibility, High concentration | 43% |
| Labriola et. al., 2009 , Denmark | Influence; information | 34% |
| Lallukka et. al., 2010, Finland | Job strain; working overtime; shift work | 39% |
| Leinonen et. al., 2011, Finland | Job control; Job demands; shift work; working overtime; workplace bullying | 41% |
| Lund et. al., 2010, Denmark | Decision authority; information | 39% |
| Månsson et. al., 1998, Sweden | Discomfort at work; shift work | 39% |
| Nilsen et. al., 2012, Norway | Concentration and attention;demanding work; job satisfaction | 32% |
| Pietilainen et. Al.,2011, Finland | Shift work; Temporary work contract; Working overtime; Job control; Job demands; social support | 41% |
| Saastamionen et. al., 2011, Finland | Social support; Job strain | 41% |
| Salo et. al., 2010, Finland | Shift work | 39% |
| Salonen et al,2003, Finland | Job stress and demands | 11% |
| Sivertsen et. al., 2006, Norway | Shift work | 34% |
| Stattin et. al., 2005, Sweden | Job control; Work demand; Social support | 45% |
| Tuomi et. al., 1991, Finland | Work load (median high/low) | 27% |
| Tuomi et. al., 1991, Finland | Mental demands; Work schedule; Social organization; Possibilities for development | 48% |
| Turner et. al., 2007, USA | Hectic work; Working very fast; Excessive work; Supervisor listen; Take breaks when wants; Satisfaction | 36% |
| van den Berg et. al., 2010,  Europe | Job control; Effort reward imbalance; Time pressure | 45% |
